# Integrated lithium niobate photonic devices for photonic quantum information science

**DOI:** 10.1186/s40580-025-00530-0

**Published:** 2025-12-31

**Authors:** Changhyun Kim, Hansol Kim, Sunghyun Moon, Hojoong Jung, Hyounghan Kwon

**Affiliations:** 1 Center for Quantum Technology, Korea Institute of Science and Technology, Seoul, Republic of Korea; 2 School of Integrated Technology, Yonsei University, Seoul, Republic of Korea; 3 Division of Quantum Information, KIST School, Korea University of Science and Technology, Seoul, Republic of Korea

## Abstract

**Abstract:**

Integrated thin-film lithium niobate (TFLN) photonics has emerged as a powerful platform for quantum information science, offering its outstanding nonlinear, electro-optic (EO), and integration capabilities. In this review, we present the latest advances in TFLN-based integrated photonics tailored to quantum technologies. We first explore state-of-the-art quantum light sources realized in both straight waveguide and resonator configuration, including high-brightness photon pair generations, squeezed light, and versatile entanglement schemes. Next, we detail progress in integrated photonic processors, with a focus on programmable interferometric networks, ultrafast EO modulators, and essential passive components for photonic qubit processing. We then address critical challenges in optical interfacing and detection technologies, discussing recent innovations in low-loss fiber-to-chip and grating coupler designs, as well as the integration of on-chip single photon detectors. This review provides a forward-looking perspective on scalable quantum photonic systems that could underpin future advances in quantum communication, computing, and sensing.

**Graphical abstract:**

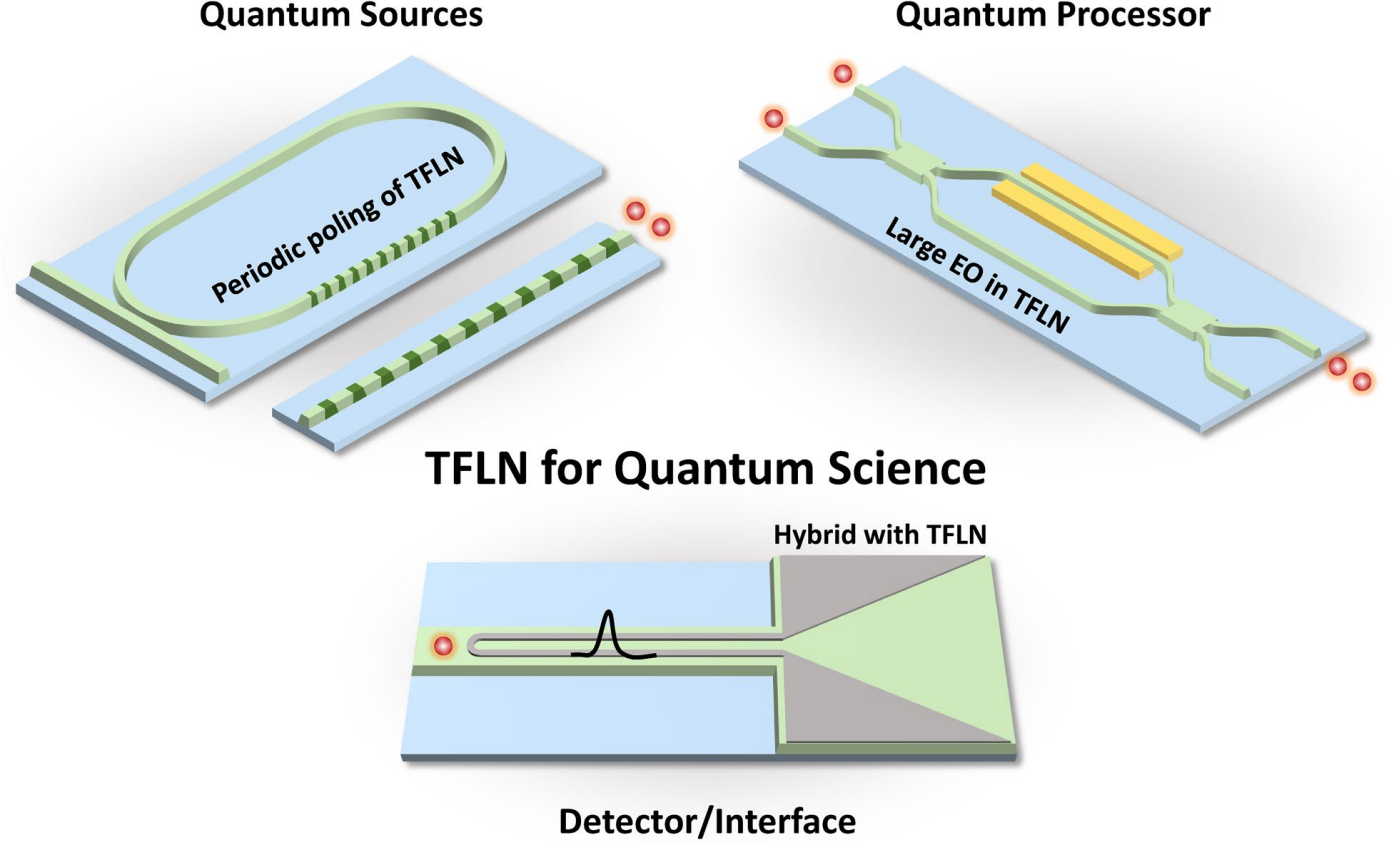

## Introduction

Quantum information science and technology utilize quantum mechanical phenomena, such as superposition, entanglement, and quantum interference, to enable innovative approaches to information processing [1, 2], secure communication [3–5], high precision sensing [6, 7], and capabilities that surpass classical computational limits [8–10]. The generation, control, transfer, and detection of quantum states are at the core of quantum information science, which requires platforms capable of reliably maintaining quantum coherence, implementing quantum logic operations, and enabling robust transport and detection of quantum information, such as trapped ions, superconductors, photons, and neutral atoms [11–16]. Among these platforms, photonic systems fulfill these requirements by exploiting inherently weak interaction with their environment even at room temperature, significantly mitigating decoherence, and allowing high-speed information processing [17]. Quantum photonic information processing exploits photons as quantum information carriers, benefiting significantly from their intrinsic coherence, large bandwidth, various degrees of freedom in photonics, and amenability to precise manipulation through linear optical interactions [18, 19]. With these advantages, quantum photonics has successfully demonstrated pivotal quantum experiments such as boson sampling [20, 21] and quantum walks [22–26], practical quantum key distribution (QKD) protocols [27–29], and quantum-enhanced sensing techniques [30, 31], reinforcing its central role in advancing practical quantum technology implementations.

Particularly, integrated photonic systems have been identified as critical components in the realization of advanced quantum photonic systems [32–35]. Integrated photonics often involve the nanofabrication of photonic structures, such as waveguides, modulators, beam splitters, and detectors onto a singular substrate, fundamentally addressing intrinsic limitations of bulk optical systems, such as mechanical instability, complex optical alignment demands, limited scalability, and environmental sensitivity. Consequently, integrated photonic circuits will facilitate unprecedented miniaturization, improved operational stability, enhanced reproducibility, and scalable fabrication processes vital for transitioning quantum photonic systems from laboratory demonstrations to commercial deployments. Silicon, silicon nitride, and III-V photonic platforms have been extensively explored and successfully implemented in quantum photonic research [32, 33, 35]. However, these traditional materials present intrinsic constraints, including limited nonlinear optical efficiencies, absent or weak intrinsic electro-optic (EO) modulation capabilities, small bandgap, non-negligible loss, and substantial integration challenges. To make these constraints clear, Table 1 summarizes representative figures of merit for silicon, silicon nitride, AlGaAs, and thin-film lithium niobate (TFLN). These limitations have spurred the pursuit of alternative integrated photonic platforms tailored explicitly to meet quantum technology's stringent performance criteria.

**Table 1:** Comparison of key figures of merit for major integrated photonic platforms [35]

Figure of merit	TFLN	Si [36, 37]	SiN [38, 39]	AlGaAs [40]
Main nonlinear coefficient	$d_{33}\sim 25$–30 pm/V	relies on $\chi ^{(3)}$	relies on $\chi ^{(3)}$	$d_{14}\sim 100$ pm/V
EO modulation	High-speed Pockels, $V_{\pi }L\sim 1$–2 V$\cdot$cm	Carrier-depletion/injection, $V_{\pi }L>10\,\hbox {V}\cdot \hbox {cm}$	No intrinsic EO effect	Native EO effect but higher loss
Typical propagation loss (telecom)	$<0.05$–0.2 dB/cm	$<0.1$ dB/cm	$<0.01$ dB/cm (ultra-low)	$<1$ dB/cm (typical)
Transparency window (nm)	350–5000	1100–4000	350–5000	800–2750 (Varies with composition)
Integration maturity	Emerging commercial foundries	Very mature CMOS ecosystem	High-volume foundries	Moderate maturity (smaller foundry base)
Detector compatibility	Heterogeneous integration of SNSPDs feasible	Mature heterogeneous integration of detectors	Compatible with heterogeneous detector integration	Monolithic integration of emitters and detectors possible

Emerging prominently among these alternative integrated platforms, TFLN has garnered significant research interest due to its exceptional material properties that are uniquely suited to quantum information science applications. TFLN exhibits an outstanding second-order nonlinear optical susceptibility ($\chi ^{(2)}$), enabling efficient nonlinear optical interactions, a remarkably large EO coefficient supporting rapid and energy-efficient modulation, a wide optical transparency range extending from visible to infrared wavelengths, and exceptionally low optical propagation losses [41–48]. These advantageous properties position TFLN to facilitate diverse quantum functionalities [49–51]. Consequently, TFLN-based integrated photonics is gaining increasing attention in quantum optical research and technology development. Although previous reviews have investigated TFLN broadly across materials and device technologies, quantum applications have typically been treated as part of larger platform overviews [41–45]. Given the rapid rise of quantum science and technology and its specific requirements for photon generation, control, and detection, there is now a clear need for a focused assessment of TFLN for quantum information science. This review addresses that need by concentrating on how this platform meets the demand of advanced quantum photonic systems.

**Fig. 1:**
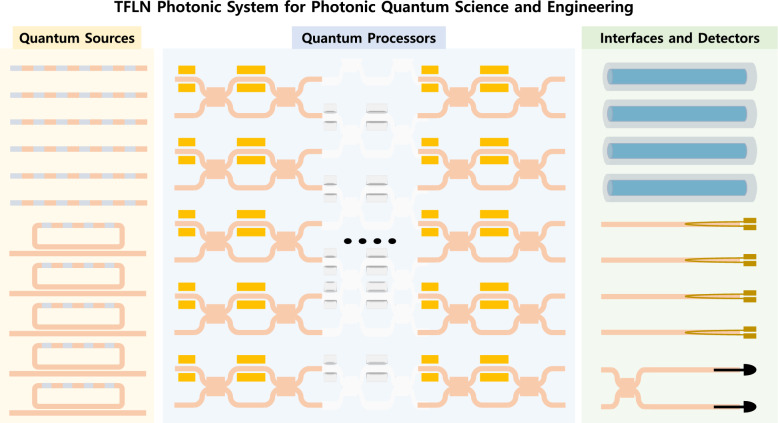
Conceptual architecture of an integrated TFLN photonic quantum system. The photonic quantum system consists of three major building blocks: probabilistic quantum sources (left), quantum processors (center), and interfaces and detectors (right). TFLN enables the efficient generation of quantum light through strong nonlinear effects and large-scale reconfigurable interferometric networks for quantum information processing. Furthermore, integrated TFLN photonic quantum systems require efficient coupling to optical fiber interfaces and on-chip detectors, forming a scalable and multifunctional quantum photonic platform

Photonic quantum systems often consist of three major building blocks: quantum state generators, quantum information processors, and efficient interfaces and detectors for quantum measurement, as illustrated in the conceptual architecture in Fig. 1. From the viewpoint of the photonic quantum system, TFLN has emerged as one of the promising candidates for integrated photonic quantum systems, offering unique advantages in both quantum light generation and large-scale quantum photonic processing [45].

First, the strong second-order nonlinearity in TFLN enables the efficient generation of quantum lights such as bright heralded single photons, high-quality multi-photon entangled states, and strongly squeezed vacuum states [45, 46]. Such quantum lights serve as the foundation for generating more complex quantum states required for advanced photonics quantum science and engineering. For example, multi-photon cluster states and non-Gaussian states are essential for universal quantum computing [52, 53].

In addition, the large EO coefficient and low propagation loss of TFLN waveguides allow for reconfigurable manipulation of quantum state manipulation, entanglement generation, and projective measurements in programmable manner [45, 46, 54, 55]. Moreover, fast photonic interferometers can serve as low-loss, multi-channel, high-speed photonic switches, which are among the most important components required for multiplexing [56, 57]. The multiplexing is indeed essential for realizing scalable quantum photonic systems that overcome the inherent probabilistic nature of photonic quantum systems [15, 58].

Finally, to realize a truly scalable photonic quantum system that integrates multiple quantum photonic chips, it is crucial to develop low-loss optical interfaces that can connect chips via optical fibers. In such architectures, optical fibers not only serve as interconnects but can also function as short-term quantum memories. Therefore, the development of highly efficient, low-loss, and mode-matched coupling schemes between TFLN chips and fiber networks is a key technological challenge. Furthermore, since photonic quantum information processing fundamentally relies on the detection of photons for quantum measurement, integrating high-performance detectors, such as single-photon detectors, photon-number-resolving detectors, and near-unity-efficiency avalanche photodiodes, on or near the chip is another critical direction for achieving fully functional and compact quantum photonic processors.

In this review, we present the current state-of-the-art in TFLN integrated photonic devices, with a particular focus on their application to quantum information science. We begin with a background overview covering the fundamental physics, device design, and fabrication processes of TFLN, including nonlinear optical effects, EO modulation, and waveguide engineering techniques. This provides the necessary foundation for understanding the capabilities and limitations of TFLN in quantum photonic applications. We then examine quantum light sources realized on the TFLN platform, highlighting photon-pair generation, squeezed light, and entangled-state sources with high brightness and fidelity. This is followed by a discussion on quantum processors, including reconfigurable interferometric architectures and high-speed EO modulators, which enable dynamic quantum state manipulation and scalable circuit operations. Next, we explore recent progress in quantum interfaces and on-chip detector integration, emphasizing efficient photon detection, fiber-to-chip coupling, and hybrid system connectivity. Finally, we conclude with an outlook on the key technological challenges and future research directions critical to advancing the scalability and commercialization of integrated quantum photonic systems based on TFLN.

## Backgrounds

In this chapter, we present a comprehensive review of the background of nonlinear dynamics, Pockels effect, design, and fabrication of TFLN. First, theoretical concepts governing nonlinear dynamics and EO modulation are introduced. Next, the design methods of components is introduced, followed by basic fabrication techniques crucial for realizing integrated photonic devices. Emphasis is placed on key nonlinear optical processes, phase-matching strategies, advanced waveguide design, precise domain engineering, and fabrication methodologies that collectively underpin the exceptional capabilities of TFLN in quantum photonic applications.

### Crystal cuts and anisotropy in TFLN

Lithium niobate is a uniaxial crystal with distinct ordinary and extraordinary refractive indices, which introduces strong anisotropy in waveguide design. At the telecom wavelength of 1550 nm, the ordinary refractive index is approximately $n_o \sim 2.21$, while the extraordinary refractive index is approximately $n_e \sim 2.14$. The wafer cut determines how these indices align with the propagation and confinement directions of the guided modes. In Z-cut TFLN, the crystal *z*-axis is normal to the wafer surface, so TM-like modes interact with the extraordinary index, while TE-like modes primarily experience the ordinary index. In X-cut TFLN, the *z*-axis lies in-plane, so the guided mode polarization projects differently onto $n_o$ and $n_e$ depending on the waveguide orientation relative to the crystal axes. Unless bendings of the waveguide are carefully designed, they can result in unwanted loss and coupling between the modes.

In X-cut devices, anisotropy also makes waveguide bending especially challenging. As waveguides curve, the varying projection of the optical field onto the crystal axes can induce polarization rotation or mode coupling. These effects are amplified in high-index-contrast geometries, where small bend radii may lead to multi-mode behavior, polarization mixing, or excess scattering. To mitigate these issues, bends in TFLN circuits must be designed with sufficiently large radii, while mode size and polarization alignment must be carefully controlled. Such optimization is essential to achieve compact yet low-loss circuits in TFLN, supporting both classical photonic integration and advanced quantum applications.

### $\chi ^{(2)}$-nonlinear dynamics of TFLN

In nonlinear optics, the optical response of a material deviates from a linear relationship with the applied electromagnetic field intensity, revealing higher order interactions under intense illumination conditions. In quantum photonic applications, the second-order nonlinear susceptibility ($\chi ^{(2)}$) of TFLN is of particular importance, enabling efficient nonlinear optical processes [41, 45–47]. Second-order nonlinear optical processes includes second-harmonic generation (SHG), spontaneous parametric down-conversion (SPDC), sum-frequency generation (SFG), and difference-frequency generation (DFG), which are essential for quantum state preparation and frequency conversion. Within the formalism of quantum optics, $\chi ^{(2)}$-nonlinear interactions can be described by an interaction Hamiltonian given by Eq. 1 [46, 59, 60]:

1 $$\begin{aligned} \hat{H}_\textrm{int} = \epsilon _0 \int _V d^3\textbf{r} \ \chi ^{(2)} E_p(\textbf{r}, t) \hat{E}_s(\textbf{r}, t) \hat{E}_i(\textbf{r}, t) + h.c. \end{aligned}$$

Here, $E_p(\textbf{r},t)$ denotes the electric field of pump light, while $\hat{E}_s(\textbf{r},t)$ and $\hat{E}_i(\textbf{r},t)$ correspond to the quantized fields of the signal and idler photons, respectively. Maximizing the efficiency of these nonlinear processes depends on the phase-matching condition, where the interacting waves propagate coherently with matched or quasi-matched phase velocities. The phase-matching condition can be expressed by Eq. 2:

2 $$\begin{aligned} \Delta k = k_p - k_s - k_i = 0 \end{aligned}$$

where $k_p$, $k_s$, and $k_i$ are the propagation constants of the pump, signal, and idler photons, respectively.

Due to the intrinsic dispersion and birefringence of lithium niobate, exact phase matching is typically challenging. Therefore, to address this, two primary approaches have been widely studied and applied to facilitate efficient second-order nonlinear processes within TFLN [45, 46, 48]. The first approach, modal phase matching (MPM), involves phase matching between different modes [48, 61–63]. The second approach, also known as quasi-phase matching (QPM), introduces a momentum vector to compensate for the momentum mismatch between interacting light waves. QPM is typically achieved through periodic poling of ferroelectric polarization domain of TFLN, modulating the nonlinear susceptibility $\chi ^{(2)}$ along the propagation direction periodically. Thus, the modified phase-matching condition is satisfied by adding an additional reciprocal lattice vector, as shown in Eq. 3:

3 $$\begin{aligned} k_p - k_s - k_i = m\frac{2\pi }{\Lambda } \end{aligned}$$

where $\Lambda$ denotes the poling period, and *m* is the integer order of QPM. Particularly, QPM is favored due to its superior frequency conversion efficiency and broad applicability, which can be finely tuned by controlling the poling period [46, 64–66]. As the poling achieves the phase matching condition, other design parameters in the waveguide geometry enable desired dispersion engineering, which is critical for advanced nonlinear and quantum optical systems [67].

The exceptional nonlinear optical properties of TFLN are further characterized by its notably high nonlinear optical coefficient, specifically the $d_{33}$ tensor component (approximately 27 pm/V), one of the highest among conventional nonlinear optical materials [45, 46]. Also, $d_{31}$, $d_{51}$, and $d_{22}$ are approximately 5.95, 5.95, and 3.07 pm/V, respectively. Efficient utilization of $d_{33}$ is strongly orientation dependent. In Z-cut TFLN, where the crystal *z*-axis is normal to the substrate, periodic poling through the film thickness directly enables QPM for TM-like modes. In addition, angled or curved waveguides can be implemented to project the guided field onto the *z*-axis, offering further flexibility in nonlinear interaction design. In X-cut TFLN, where the *z*-axis lies in-plane, exploiting $d_{33}$ requires careful alignment of the guided mode with the in-plane *z*-axis, typically achieved by rotating the waveguide orientation and electrodes relative to the crystal axes in combination with lateral poling. Despite these orientation-dependent challenges, the efficient nonlinear optical processes achievable with such substantial nonlinearity are further augmented by the extensive optical transparency range of lithium niobate, spanning wavelengths from ultraviolet through visible to mid-infrared (approximately 350 nm - 5 $\mu$m). This spectral versatility makes TFLN exceptionally suited to quantum photonic applications across diverse frequency bands, facilitating quantum networks operating in visible, near-infrared (NIR), and telecommunication wavelengths.

### Pockels effect of TFLN

The Pockels effect, or also called as linear EO effect, is observed in TFLN due to non-centrosymmetric crystal. TFLN offers significant advantages for integrated photonics due to its high EO coefficient, which allows pronounced modulation effects even under low electric fields [41, 44, 45, 68, 69]. The thin-film architecture supports high index contrast in waveguides, consisting of a $\hbox {LiNbO}_3$ layer ($n \sim 2.2$ at 1550 nm) on a $\hbox {SiO}_2$ substrate ($n \sim 1.44$), supports a large core-cladding index contrast of about $\sim$ 0.7, leading to compact modal area, small gap size between electrodes, and minimal operating voltage. These characteristics, combined with the capability for high-speed operation (often in the GHz range), make TFLN an attractive platform for next-generation photonic integrated circuits.

The fundamental relation is often expressed as a change in the inverse square of the refractive index:

4 $$\begin{aligned} \Delta \left( \frac{1}{n^2}\right) = rE \end{aligned}$$

where *r* is the EO coefficient and *E* is the external electric field. In the TFLN platform, most commonly exploited and largest coefficient is $r_{33}$ for the extraordinary axis. For small perturbations, this can be approximated by:

5 $$\begin{aligned} \Delta n = \frac{1}{2} n^3r_{33}E \end{aligned}$$

where the refractive index is modulated by the applied electric field. When light propagates through a TFLN waveguide, the induced refractive index change results in a corresponding phase shift. In practical EO modulators, this phase shift can be converted into amplitude modulation, often through a Mach-Zehnder interferometer configuration, which enables the modulation of light intensity in response to an electrical signal. Also, for quantum optical applications, direct control of the optical phase is equally essential, as it determines interference and the full quantum state of photons.

A further consideration for EO modulation is device stability under cooling. LN is a pyroelectric material, and depending on the wafer cut, cooling can generate internal electric fields that disturb device operation. This poses challenges for cryogenic quantum applications. Nonetheless, recent experiments have demonstrated that with careful device design and packaging, TFLN modulators can remain DC-stable even at liquid nitrogen temperatures (77 K), showing promise for cryogenic-compatible operation in quantum systems [70].

While this part focuses on the Pockels effect as the primary EO mechanism in TFLN, it is worth noting that other modulation schemes are also feasible in this platform [69, 71, 72]. In particular, acousto-optic modulation, based on the piezoelectric properties of lithium niobate, enables dynamic control of light through surface acoustic waves [71]. Additionally, thermo-optic modulation, which relies on temperature-induced changes in refractive index, is widely observed across many photonic materials and, despite its relatively slow modulation speed, is also applicable to TFLN [72]. These alternative mechanisms offer complementary functionalities and can be leveraged alongside the Pockels effect to expand the versatility of TFLN-based integrated photonic devices [71, 72].

### Methods for components design

Designing TFLN integrated photonic components, including those on the TFLN platform, follows the general principles of electromagnetic simulation employed across many photonic material systems. Tools such as the finite-difference time-domain (FDTD) method, finite element method (FEM), and eigenmode expansion (EME) are widely used to simulate light propagation and modal behavior within complex waveguide geometries. However, LN-based devices exhibit several material- and process-specific characteristics that must be carefully accounted for during the design stage. In particular, LN has high birefringence, which strongly affects the polarization behavior of guided modes. Furthermore, due to fabrication challenges, discussed in more detail in the following section, TFLN waveguides often have slanted sidewalls of 50°–80° or asymmetric cross-sections. These features can lead to unwanted effects such as polarization mode conversion between TE and TM modes, as well as increased bending loss in waveguide circuits. Accurate modeling of these factors is essential to prevent performance degradation and to ensure reliable operation of the final integrated photonic device.

**Fig. 2:**
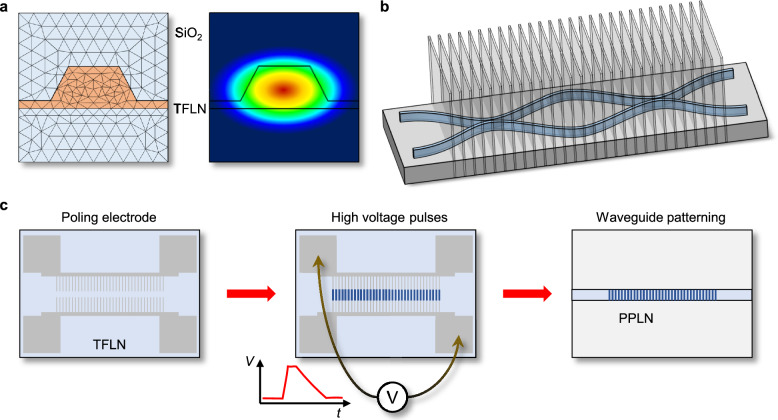
Simulation and periodic poling fabrication. **a** Numerical modeling illustrating the electromagnetic simulation of TFLN waveguide cross-section using FEM and **b** EME for analyzing mode behavior and propagation within photonic circuits. **c** Fabrication steps depicting periodic poling of TFLN, involving the application of spatially modulated high-voltage pulses to induce ferroelectric domain inversion, thus forming periodically poled lithium niobate (PPLN) structures necessary QPM

FDTD, FEM, and EME play distinct roles in the design of TFLN photonic components. FDTD is well-suited for modeling broadband and time-dependent phenomena, such as pulse propagation and dynamic switching, though it can be computationally intensive for 3D structures. FEM is widely used for frequency-domain analysis, offering high accuracy in mode solving and resonant behavior (Fig. 2a), especially in complex geometries. EME provides an efficient approach for simulating mode evolution in structures with slowly varying cross sections (Fig. 2b), such as tapers and couplers, but is less accurate in regions with abrupt structural changes. By selecting the appropriate tool based on the device geometry and target phenomena, designers can achieve accurate and efficient modeling of TFLN integrated photonic circuits.

Inverse design techniques optimize photonic structures by directly targeting specified optical performance [73, 74]. Unlike conventional methods, inverse design automates geometry adjustments based on defined performance metrics. Inverse design begins by establishing a clear figure of merit (FOM), $F(\textbf{p})$, depending on geometric parameters, where *F* can be calculated through the electromagnetic simulations. Then, the optimization problem can be described as:

6 $$\begin{aligned} \textbf{p}^*=\arg \min _{\textbf{p}} F(\textbf{p}) \end{aligned}$$

For optimization, gradient-descent method are commonly exploited [75]. Gradient of the parameters can be obtained through the adjoint variable methods or automatic differentiation. Also, inverse design can incorporate fabrication constraints, such as minimum feature size, which can produce practical and fabricable designs [62, 63, 76, 77]. The inverse design method often identifies unconventional structures with superior performance, extending the range of possible designs for integrated photonic devices. Nevertheless, inverse design can be computationally expensive due to the numerous iterations required to converge on an optimal solution. Additionally, the resulting designs might sometimes be challenging to fabricate or may require further refinement to meet practical constraints, and there is always a risk of converging to local minima if the optimization landscape is not well managed.

### Basic fabrication of TFLN integrated photonics

Fabrication techniques for TFLN photonic integrated circuits have markedly evolved, solidifying TFLN as a leading material platform within integrated quantum photonics. The integration of quantum functionalities necessitates precise control over surface quality, device geometry, and uniformity across wafer scales [41, 42]. Achieving these stringent conditions has become feasible through significant advancements in material growth, wafer bonding, ion slicing, precision lithographic techniques, and advanced etching processes [41, 42].

The initial preparation of TFLN substrates is based on a wafer bonding and ion-slicing process, widely known as the Smart Cut method [78]. In this technique, bulk lithium niobate wafers are subjected to hydrogen-ion implantation to create a controlled subsurface damage layer at a predetermined depth, followed by bonding to an insulating handle substrate, typically a silicon wafer with a thermally grown silicon dioxide layer ($\hbox {SiO}_2/\hbox {Si}$). A subsequent thermal annealing step induces splitting along the implantation-induced damage layer, resulting in a thin, high-quality lithium niobate film uniformly transferred to the handle wafer. After layer transfer, a chemical–mechanical polishing process is commonly employed to remove implantation-related surface damage and achieve the required surface smoothness and planarity for the fabrication of low-loss photonic devices.devices. Key process parameters, including implantation dose, ion energy, annealing temperature, and poling conditions, must be carefully optimized, as they significantly influence film crystallinity, defect density, thickness uniformity, and ultimately optical propagation characteristics [78]. The resulting TFLN typically has a thickness ranging from a few hundred nanometers to several micrometers.

Ferroelectric domain inversion through periodic poling is optionally performed when QPM is required to utilize the second-order nonlinear effects [41–44, 46]. Periodic poling facilitates nonlinear optical processes by compensating phase mismatches, thereby significantly enhancing conversion efficiencies critical in quantum optical applications such as photon pair generation. Periodic poling involves the controlled inversion of ferroelectric domains within the lithium niobate crystal lattice, typically accomplished by applying spatially modulated high-voltage electric field pulses (Fig. 2c). High-resolution electron-beam lithography (EBL) or advanced photolithography defines electrode patterns for domain inversion, transferred subsequently to metallic electrodes via lift-off or selective etching techniques. Inverted domain structures are often characterized by using second-harmonic microscopy [64, 79, 80].

After ferroelectric domain conversion (when applicable), the waveguide pattern is written by photolithography or EBL, followed by etching techniques, predominantly inductively coupled plasma reactive-ion etching (ICP-RIE) [41, 42]. Because of challenges in chemical etching of lithium niobate, the etching process relies heavily on physical ion bombardment. As a result, achieving high selectivity between the etch mask and the substrate is challenging, and process parameters must be carefully tuned to reduce roughness and scattering loss. To minimize these losses, most LN waveguides are etched with slanted sidewalls, a characteristic that must be considered during design.

Further integration of active and passive quantum components, such as EO modulators, superconducting nanowire single-photon detectors (SNSPDs), and quantum emitter interfaces, involves additional deposition and patterning processes [56, 68, 81, 82]. Techniques such as atomic layer deposition (ALD), plasma-enhanced chemical vapor deposition (PECVD), electron-beam evaporation, and sputtering are employed to produce uniform, high-quality dielectric and conductive thin films essential for efficient EO modulation, photon detection, and hybrid device integration. Superconducting nanowire detectors, frequently utilizing materials such as niobium nitride (NbN) or niobium titanium nitride (NbTiN), are integrated through ultra-high vacuum deposition of a few-nanometer thick superconducting film and careful nano-scale patterning, essential for achieving high detection efficiencies and maintaining superconducting performance [81, 82].

## Quantum light sources

Harnessing its intrinsic $\chi ^{(2)}$ properties, extensive research has been dedicated to manipulating various quantum light states, including photon pair generation, entangled photon generation, squeezed state generation, and the creation of coherent spectral components. Notably, the exceptional ability of TFLN to confine light within dimensions as small as a few $\mu m^2$ positions it as an efficient and promising platform for strong photon-photon interactions, particularly in the form of parametric processes. In general, TFLN nanofabrication is inherently more complex than in mature platforms such as SiN or SOI, due to the challenges of achieving smooth and reproducible deep etching. Nevertheless, TFLN offers a rich degree of freedom in waveguide geometries including height, etch depth, and width which, although coupled with the complexity of nanofabrication, enables versatile dispersion engineering and phase-matching of $\chi ^{(2)}$ processes. Recent advances in fabrication techniques have steadily improved reproducibility and optical loss, making this design flexibility increasingly accessible. These remarkable features of TFLN make it a highly desirable platform for light state generation, especially in the context of non-classical light sources.

In this chapter, we review the generation of quantum light states within TFLN waveguides. We begin by briefly discussing the two primary configurations of TFLN waveguides: straight waveguides and resonators. Our focus is especially on SHG, i.e., parametric up-conversion processes, for both configurations. For the micro-cavity configuration, we also highlight the optical parametric oscillator (OPO), paying particular attention to its threshold. We explore the conversion efficiency, which serve as key metrics for determining whether TFLN is a suitable platform for quantum light sources. Lastly, we examine the application of TFLN in the generation of quantum states of light.

### System configurations: straight waveguide and microring resonator

TFLN devices commonly use X-cut and Z-cut orientations, and the choice of cut significantly influences the accessible nonlinear tensor elements, phase-matching strategies, and device integration. By appropriately selecting the orientation, a variety of desirable optical properties can be achieved. X-cut devices allow TE modes to directly access the strongest nonlinear tensor element $d_{33}$, making them particularly favorable for efficient QPM and EO modulation with in-plane electrodes, while Z-cut devices enable TM modes to utilize $d_{33}$. These distinctions extend further to resonator performance, fabrication maturity, and integration schemes. To clearly illustrate these trade-offs, Table 2 summarizes the advantages and disadvantages of the two orientations.

**Table 2:** Comparison of X-cut and Z-cut TFLN waveguides for nonlinear photonics and EO modulation

Aspect	X-cut TFLN	Z-cut TFLN
Nonlinear coefficient access	$d_{33}$ via TE modes	$d_{33}$ via TM modes
SHG efficiency	2600–$4600\%\hbox {W}^{-1}\hbox {cm}^{-2}$ [83–85] up to $10^4\%\hbox {W}^{-1}$ with adaptive poling [86]	$1900\%\hbox {W}^{-1}\hbox {cm}^{-2}$ [87] $1470\%\hbox {W}^{-1}\hbox {cm}^{-2}$ with subwavelength poling [88]
Resonator performance	High-*Q* up to $10^5$–$10^7$ [89, 90]	up to $10^6$ [66]
EO modulation	Efficient $r_{33}$ access via in-plane field, low $V_\pi L$	$r_{33}$ accessible via out-of-plane field; complex electrode design and lower efficiency
Challenges	Sensitivity to film inhomogeneity	Electrode placement and fabrication complexity

#### Straight waveguide

**Fig. 3:**
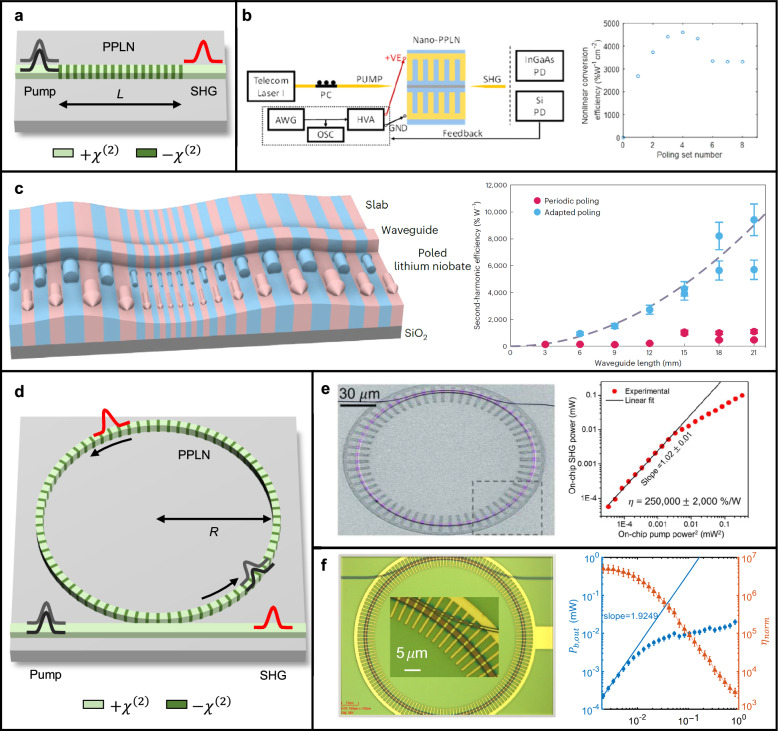
Source system configurations on PPLN waveguides. **a** Straight-waveguide configuration illustrating the single-pass interaction between pump and SH fields. **b** Straight PPLN waveguide designed through an actively monitored poling method, achieving an exceptionally high normalized conversion efficiency. Reproduced with permission [64]. Copyright 2019, Optica Publishing Group. **c** Adaptively poled TFLN waveguide with optimized poling conditions along the entire device length, substantially reducing impacts from device inhomogeneities. Reproduced with permission [86]. Copyright 2024, Springer Nature. **d** Microring resonator configuration with multiple circulations of pump and SH fields. **e** Doubly-resonant Z-cut PPLN-MR resonator demonstrating efficient frequency conversion. Reproduced with permission [65]. Copyright 2019, Optica Publishing Group. **f** Advanced optimization of nonlinear coupling strength ($g_0$) within a doubly-resonant Z-cut PPLN-MR resonator. Reproduced with permission [66]. Copyright 2020, Optica Publishing Group

In a straight TFLN waveguide (Fig. 3a), the evolution of normalized pump and second harmonic (SH) field envelopes, denoted as $A_{\textrm{pump}}$ and $A_{\textrm{SH}}$, respectively, can be described by a set of coupled-mode Eqs. 7 and 8 [46, 60]:

7 $$\begin{aligned} \frac{dA_{\textrm{pump}}(z)}{dz}= & -i \kappa A_\textrm{pump}^*(z) A_{\textrm{SH}}(z) e^{-i \Delta k z} \end{aligned}$$

8 $$\begin{aligned} \frac{dA_{\textrm{SH}}(z)}{dz}= & -i \kappa A_\textrm{pump}(z)^2 e^{i \Delta k z} \end{aligned}$$

Here, $\kappa$ and $\Delta k$ represent the nonlinear coupling coefficient between the pump and SH fields, and the phase mismatch, respectively [46]. The nonlinear coupling coefficient $\kappa$ can be expressed as,

9 $$\begin{aligned} \kappa = \frac{(2Z_0)^{1/2}\omega d_{eff}}{cn_{\textrm{pump}} (n_{\textrm{SH}}A_{eff})^{1/2}} \end{aligned}$$

where $Z_0$ is the free-space impedance, $d_{eff}$ is the effective nonlinear coefficient, $A_{eff}$ is the effective area, *c* is the speed of light, $\omega$ is the angular frequency, and $n_{\textrm{pump}}$ and $n_{\textrm{SH}}$ are the refractive indices at the pump and SH frequencies, respectively [46]. From the normalized field envelopes, the pump and SH powers are given by $P_{\mathrm {pump(SH)}}(=|A_{\mathrm {pump(SH)}}|^2)$. Then, when the pump power is sufficiently higher than the SH power, integrating Eq. 8 over the periodically poled length, $L_{\textrm{pp}}$, yields the output SH power as,

10 $$\begin{aligned} P_{\textrm{SH}} = \kappa ^2 P_{\textrm{pump}}^2 L^2_{\textrm{pp}} \textrm{sinc}^2\left( \Delta kL_{\textrm{pp}}/2\right) \end{aligned}$$

When there is no phase mismatch between pump and SH light, that is, $\Delta k=0$, the output SH power exhibits a quadratic dependence on the square of the pump power, with the normalized conversion efficiency, $\eta _0=\kappa ^2$, serving as a proportional coefficient.

11 $$\begin{aligned} P_{\textrm{SH}} = \kappa ^2 P_{\textrm{pump}}^2 L^2_{\textrm{pp}} = \eta _0 P_{\textrm{pump}}^2 L^2_{\textrm{pp}} \end{aligned}$$

Since the effective area is proportional to the square of the mode area of the pump multiplied by the mode area of the SH light, i.e., $A_{eff} \propto A_{\omega }^2 A_{2\omega }$ [46], the coupling coefficient $\kappa$ is inversely proportional to the mode areas of the pump and SH light as $\kappa \propto A_{\textrm{pump}}^{-1} A_{\textrm{SH}}^{-1/2}$. As a result, the small size of the TFLN waveguide, down to the order of a few $\mu \textrm{m}^2$, leads to enhancement in SHG conversion efficiency. In particular, TFLN-based SHG can achieve one to two orders of magnitude higher normalized conversion efficiency compared to bulk lithium niobate-based SHG.

Among extensive research on SHG in periodically poled TFLN, a pioneering periodically poled TFLN device compatible with photonic integrated circuits has been achieved in 2016 [91]. In this study, by burying the LN beneath a silicon nitride layer, proposed TFLN device exhibits a similar mode area for both the pump and SH fields, resulting in a high mode overlap. This configuration has led to a normalized conversion efficiency of 160%$\textrm{W}^{-1}\textrm{cm}^{-2}$, which is an order of magnitude higher than that of bulk periodically poled LN [92–94]. Subsequently, in 2018, a one-order-higher normalized conversion efficiency of 2600%$\textrm{W}^{-1}\textrm{cm}^{-2}$ has been achieved in a 4 mm length X-cut periodically poled TFLN device, attributed to high optical confinement, a large overlap between the pump and SH modes, and high fidelity in first-order periodic poling [83]. The observed discrepancy between the experimentally measured normalized conversion efficiency and the theoretically predicted value of 4500%$\textrm{W}^{-1}\textrm{cm}^{-2}$ was attributed to inhomogeneities within the device. In the following year, a noteworthy study has reported a normalized conversion efficiency of 4600%$\textrm{W}^{-1}\textrm{cm}^{-2}$ within a 0.3 mm long X-cut periodically poled TFLN device, the highest achieved to date for periodic poling on single layer waveguides(Fig. 3b) [84]. This exceptional efficiency was attained using an iterative, reversible, and actively monitored approach to the periodic poling process [64]. In 2020, a normalized conversion efficiency of $3757\%\hbox {W}^{-1}\hbox {cm}^{-2}$ has been demonstrated in a 5-mm-long TFLN device employing a shallow-etched waveguide geometry, which enhanced fabrication tolerance by achieving width sensitivity similar to unetched LN structures, while preserving the high efficiency of etched waveguides [85].

Despite these impressive advancements, achieving simultaneously long interaction lengths and high conversion efficiencies has remained challenging. This difficulty primarily stems from the extreme sensitivity of periodically poled TFLN devices to local inhomogeneities, combined with the intrinsic non-uniformity of the TFLN films, which has created significant challenges in realizing long and efficient devices [95]. Then, in 2024, a groundbreaking study introducing an adaptive poling technique (Fig. 3c), which is designed to optimize poling conditions and parameters to ensure phase-matching conditions across the entire poling length with minimal impact from device inhomogeneities, has demonstrated a conversion efficiency of $\sim$ $10^4$ %$\textrm{W}^{-1}$ in a 21 mm X-cut TFLN device [86].

Unlike X-cut TFLN, where the transverse electric (TE) mode experiences the highest nonlinear coefficient, i.e., $d_{33}$, the transverse magnetic (TM) mode exhibits the highest nonlinear coefficient in Z-cut TFLN waveguides. In 2020, tunable SHG was achieved in a Z-cut TFLN device with a normalized conversion efficiency of 2,400%$\textrm{W}^{-1}\textrm{cm}^{-2}$ [87]. By combining group-velocity mismatch (GVM) engineering with thermal tuning, efficient SHG operation was demonstrated over a wide wavelength range from 1530 nm to 1583 nm. More recently, in 2024, symmetric SHG was reported in a sub-wavelength 370 nm periodic poling within a 6 mm long Z-cut waveguide, achieving an efficiency of 1,470 %$\textrm{W}^{-1}\textrm{cm}^{-2}$ [88]. This advancement highlights the capability of fabricating low-loss TFLN waveguides with sub-wavelength poling precision, and opens up new opportunities for implementing efficient backward SPDC photon sources. Beyond SHG studies, ultrabroadband nonlinear frequency conversions in dispersion engineered periodically poled TFLN was demonstrated in 2020 [67]. This work utilized the synergy between dispersion tailoring in nanophotonic structures and engineered QPM in TFLN, allowing ultrafast pulses to undergo highly efficient, broadband frequency conversion under quasi-static interaction conditions. Two years later, dispersion engineering enabled intense parametric amplification within a chip-scale waveguide device [96]. That same year, quasi-static parametric amplification was achieved, delivering a high parametric gain of 71 dB with a few pJ of input power [97]. These advancements have significantly propelled the development of nonlinear light sources at the nanoscopic level.

#### Microring resonator

In the resonator configuration (Fig. 3d), the generated SH power can be expressed as [65],

12 $$\begin{aligned} P_{\textrm{SH}} = \frac{g_0^2}{\hbar } \cdot \frac{\omega _{\textrm{SH}}}{\omega _{\textrm{pump}}^2} \cdot \frac{2\kappa _{c,\textrm{SH}}}{\delta _{\textrm{SH}}^2 + \kappa _{\textrm{SH}}^2} \cdot \left( \frac{2\kappa _{c,\textrm{pump}}}{\delta _{\textrm{pump}}^2 + \kappa _{\textrm{pump}}^2} \right) ^2P_{\textrm{pump}}^2 \end{aligned}$$

Here, $\hbar$ represents the reduced Planck constant. The parameters $\kappa$, $\kappa _c$, and $\delta$ correspond to the total decay rate, the coupling decay rate, and the detuning from resonance, respectively. The total decay rate is given by $\kappa = \kappa _c + \kappa _i$, where $\kappa _i$ is the intrinsic loss rate. Subscripts indicate whether the quantities are associated with the pump mode or the SH mode. Under critical coupling conditions, where $\kappa _c = \kappa _i$, and assuming perfect resonance ($\delta _{\textrm{pump}} = \delta _{\textrm{SH}} = 0$), the SH output power can be expressed as,

13 $$\begin{aligned} P_{\textrm{SH}} = \frac{g_0^2}{\hbar } \cdot \frac{1}{\omega _{\textrm{pump}}^4} \cdot \frac{\omega _{\textrm{SH}}}{2\kappa _{i,\textrm{SH}}} \cdot \left( \frac{\omega _{\textrm{pump}}}{2\kappa _{i, \textrm{pump}}} \right) ^2P_{\textrm{pump}}^2 \end{aligned}$$

Furthermore, using the relationship between the intrinsic decay rate and the intrinsic quality factor (Q-factor) $Q_i$,

14 $$\begin{aligned} Q_{i,\mathrm {pump(SH)}}=\frac{\omega _{\mathrm {pump(SH)}}}{2\kappa _{i,\mathrm {pump(SH)}}} \end{aligned}$$

the SH power can be rewritten as,

15 $$\begin{aligned} P_{\textrm{SH}} = \frac{g_0^2}{\hbar \omega _\textrm{pump}^4} \textrm{Q}_{i,\textrm{SH}}\textrm{Q}^2_{i,\textrm{pump}}P_\textrm{pump}^2 = \frac{8g_0^2}{\hbar \omega _\textrm{pump}^4} \textrm{Q}_{L,\textrm{SH}}\textrm{Q}^2_{L,\textrm{pump}}P_\textrm{pump}^2 \end{aligned}$$

where $Q_{L,\mathrm {pump(SH)}}$ denotes the loaded Q-factor of the respective modes. It is worth noting that, unlike in a straight waveguide where light propagates through the TFLN device in a single-pass, in a resonator, the light circulates inside the resonator multiple times. As a result, normalizing by a practical poling length is not straightforward. Therefore, the SHG characteristic of a TFLN resonator is identified with the supplied pump power and the generated SH power in terms of conversion efficiency, $\eta _{R}$, which is written as,

16 $$\begin{aligned} \eta _{R} = \frac{P_{\textrm{SH}}}{P_\textrm{pump}^2} = \frac{8g_0^2}{\hbar \omega _\textrm{pump}^4}\textrm{Q}_{L,\textrm{SH}} \textrm{Q}^2_{L,\textrm{pump}} \end{aligned}$$

The conversion efficiency of the resonator strongly depends on the Q-factors of pump and SH light, and to maximize the enhancement factor $\textrm{Q}^2_{\textrm{pump}}\textrm{Q}_{\textrm{SH}}$, extensive research has been conducted on the design of high-Q resonators, ultimately achieving intrinsic Q-factor up to $10^7$ [89, 98–104].

In 2019, using a microdisk with high Q-factors for both pump and SH light, a conversion efficiency of $\sim$ 9900%$\textrm{W}^{-1}$ has been achieved, and the combination of a high-Q factor and QPM conditions for higher harmonics even enabled third-harmonic generation (THG) through a cascaded frequency conversion process [105]. In the same year, a doubly-resonant configuration achieved an impressive conversion efficiency of 230,000%$\textrm{W}^{-1}$ [106]. In this study, an X-cut TFLN resonator was employed to harness the highest nonlinear coefficient for fundamental TE modes in both the pump and SH components. A similar investigation, also utilizing a doubly-resonant scheme but leveraging a Z-cut resonator (Fig. 3e), was reported in the same year [65]. This approach facilitated efficient frequency conversion between the fundamental TE pump mode and the fundamental TM SH mode, achieving an even higher conversion efficiency of 250,000%$\textrm{W}^{-1}$. Notably, in both studies, the Q-factors for the pump and SH light exceeded $10^5$, underscoring the high optical confinement and minimal loss in these resonators. In 2020, further optimization of the nonlinear coupling strength $g_0$ in the doubly-resonant Z-cut TFLN device achieved a remarkable conversion efficiency of 5,000,000%$\textrm{W}^{-1}$ (Fig. 3f), which is 20 times higher than the result reported in the previous year [66]. In 2024, a study reported a high SH conversion efficiency of 149,000%$\textrm{W}^{-1}$, using modal phase matching (MPM) between a fundamental TE pump and SH $\hbox {TE}_{01}$ modes [90]. This was achieved in a microring resonator fabricated on an X-cut dual-layered TFLN platform, where two LN layers with opposite c-axis orientations were bonded together. The reversed polarity disrupts the symmetry of the $\chi ^{(2)}$ nonlinearity, enabling efficient phase matching between the $\hbox {TE}_{00}$ mode in the pump band and the $\hbox {TE}_{01}$ mode in the SH band. Although this efficiency is relatively lower than that of QPM-based devices, it remains remarkably high, particularly considering that MPM can be implemented without the need for a poling process.

Table 3 benchmarks representative SHG conversion efficiencies and Q-factors across integrated platforms. The data highlight both the strong potential of TFLN and the practical impact of fabrication limited Q-factor and poling uniformity, alongside comparable constraints in AlN, GaAs/AlGaAs, GaP, $\hbox {Si}_3\hbox {N}_4$, and SOI.

**Table 3:** Summary of on-chip SHG performance across integrated platforms

Platform	Waveguide	Resonator	*Q* Factor
TFLN	2600* [83] 4600* [84]; 3757* [85] $\sim 10^4$ [86]	149,000 [90] 250,000 [65] 5,000,000 [66]	$\sim 10^5$–$10^7$ [65, 89, 98, 100, 101, 103]
AlN	–	2500 [107] 17,000 [108]	$\sim 10^5$–$10^6$ [107, 108]
(Al)GaAs	$\sim$ 40 [109]	–	$>10^6$ [110, 111]
GaP	0.4 [112]	400 [113]	$\sim 10^4$
$\hbox {Si}_3\hbox {N}_4$ (Induced $\chi ^{(2)}$)	–	2500 [114]	$\sim 10^6$ – $10^7$ [39, 114–116]
Si (Induced $\chi ^{(2)}$)	$\sim$ 13 [117]	–	$\sim 10^6$ [118]

Efficiencies are shown as normalized (% $\hbox {W}^{-1}\,\hbox {cm}^{-2}$, marked as *) or absolute (% $\hbox {W}^{-1}$ or $\hbox {W}^{-1}$)

Single-pass waveguides have reached normalized efficiencies of 2,600–4,600 %$\hbox {W}^{-1}\hbox {cm}^{-2}$, with record values approaching $10^{4}\,\%\hbox {W}^{-1}$ in long devices with adaptive poling. Resonator-enhanced devices have demonstrated cavity efficiencies exceeding $10^{5}\,\%\hbox {W}^{-1}$, with recent reports of up to $5\times 10^{6}\,\%\hbox {W}^{-1}$.

For comparison, platforms such as AlN, GaP, and GaAs/AlGaAs have reported cavity-enhanced SHG efficiencies in the range of $10^{3}$–$10^{4}\,\%\hbox {W}^{-1}$, while induced $\chi ^{(2)}$ $\hbox {Si}_3\hbox {N}_4$ devices typically remain below $\sim 10^{3}\,\%\hbox {W}^{-1}$. Although TFLN clearly benefits from the large $\chi ^{(2)}$ coefficient and correspondingly higher efficiencies, fabrication limits especially sidewall scattering, film non-uniformity, and poling precision restrict the practically attainable *Q*-factors and conversion efficiencies compared to the theoretical predictions. These constraints explain the discrepancy between calculated and measured efficiencies in long poled devices, underscoring the central role of fabrication maturity in defining the performance ceiling of TFLN photonics.

Studies on OPO based on $\chi ^{(2)}$ in a chip-scale were widely conducted on whispering gallery micro-disk and ring resonators in an early stage [119–122]. While whispering gallery micro-disk resonators capable of operating at thresholds as low as a few $\mu \textrm{W}$ have been reported [119], they typically exhibit threshold powers ranging from a few to a few tens of milliwatts [120–122]. From the perspective of nanophotonic scalability and compatibility with additional components, TFLN waveguide could be a great candidate that can overcome the challenges that whispering gallery microresonator face. In 2021, a groundbreaking demonstration was achieved using a Z-cut TFLN microcavity. By pumping the fundamental TM mode, the light experienced the nonlinear coefficient while maintaining a high-Q factor exceeding $10^5$. This enabled the realization of an ultra-low-threshold OPO at just 30 $\mu \textrm{W}$ [123]. Building on this progress, the following year saw another significant achievement with an X-cut TFLN microresonator, where OPO operation was realized at a low threshold of $\sim$ 73 $\mu \textrm{W}$ [124]. Notably, in this experiment, SH light, which generated through a telecom-band pump, was itself repurposed as a pump for the OPO, leading to the generation of telecom-band signals. This cascaded process was successfully demonstrated, marking a crucial advancement in the field. Also, the investigations on the OPOs have drawn significant attention because operating an OPO below its oscillation threshold enables the generation of nonclassical light, including squeezed and entangled states, directly on a photonic chip.

Furthermore, extensive research has been conducted on OPO comb generation and mid-infrared OPO-based spectroscopy, driving significant advancements in these fields [51, 125, 126]. Although this paper focuses on the second order nonlinear effect, we should note that researchers have actively explored Kerr comb generation and ultra-fast pulse formation, leveraging the third order nonlinear susceptibility ($\chi ^{(3)}$), dispersion engineering, and EO modulation capabilities of TFLN resonators [127–129].

These advancements in classical nonlinear optics collectively highlight the exceptional potential of TFLN for achieving highly efficient nonlinear optical processes at the chip scale, enabling compact and densely integrated photonic circuits. Such capabilities are particularly crucial for quantum photonics, where efficient nonlinear interactions are essential for the generation and manipulation of quantum states, thus positioning TFLN as a key platform for advancing quantum information processing and quantum metrology.

### Quantum states of light generation

With their strong photon-photon interactions enabled by $\chi ^{(2)}$ nonlinearities, TFLN waveguides have garnered considerable interest as a promising platform for quantum light generation. This growing attention is driven by their remarkable success over the past decade in utilizing nonlinear effects for efficient light generation, as discussed in the previous section. Their ability to facilitate robust nonlinear interactions has placed them at the forefront of quantum photonics research. In this section, we review the quantum states of light generation within TFLN waveguides.

#### Entangled photon-pair generation

Using parametric down-conversion, one pump photon can spontaneously generate a pair of photons, commonly referred to as signal and idler photons, through the conservation of energy and momentum. In the specific case of SPDC, the process is initiated by vacuum fluctuations, which introduce intrinsic quantum randomness into the generated photon pairs [130, 131]. This makes SPDC a foundational mechanism for producing correlated photon pairs and entangled photon states. Notably, the conservation of energy in the SPDC process results in strong frequency correlations between the signal and idler photons, leading to entanglement in the frequency domain [132, 133]. Periodically poled TFLN waveguides are particularly attractive platforms for photon-pair generation. In this section, we review the generation of photon pairs and entangled photon pairs in TFLN waveguides, focusing on key parameters that characterize their quantum properties.

Over the past few years, substantial progress has been made in demonstrating high-performance correlated and entangled photon-pair sources based on TFLN waveguides [134–138]. Improvements in device geometry, poling quality, loss, and dispersion engineering have led to significant advances in brightness, heralding efficiency, spectral purity, and two-photon interference visibility. In what follows, we review key experimental milestones in photon-pair and two-photon entangled state generation using TFLN platforms, highlighting representative performance metrics such as coincidence-to-accidental ratio (CAR), generation rate, spectral bandwidth, and heralded second-order correlation. These results demonstrate the rapid evolution of TFLN as a viable and competitive platform for scalable quantum light sources.

For the configuration of TFLN devices for the generation of correlated and entangled photon pairs, while resonator configurations hold the potential for enhancing nonlinearity, achieving a doubly-resonant condition between the pump and signal (or triply-resonant if the idler is also involved in the case of non-degenerated process) is essential for optimal performance [65, 66, 106]. This requires careful tuning and control of the resonant frequencies, which is inherently challenging. As a result, most studies on photon-pair generation to date have utilized straight waveguide configurations.

Additionally, when comparing X-cut to Z-cut, the X-cut offers advantages in terms of the poling process, particularly in electrode positioning and temperature control during poling fabrication. These factors make the X-cut TFLN more preferred choice to implement photon-pair generation device. Furthermore, X-cut waveguides allow for type-0 phase-matching between fundamental TE modes, promoting efficient SPDC. Consequently, extensive research has been carried out on correlated and entangled photon-pair generation in X-cut straight TFLN waveguides, exploring various geometry optimizations and the characteristic of the photon-pair produced.

**Fig. 4:**
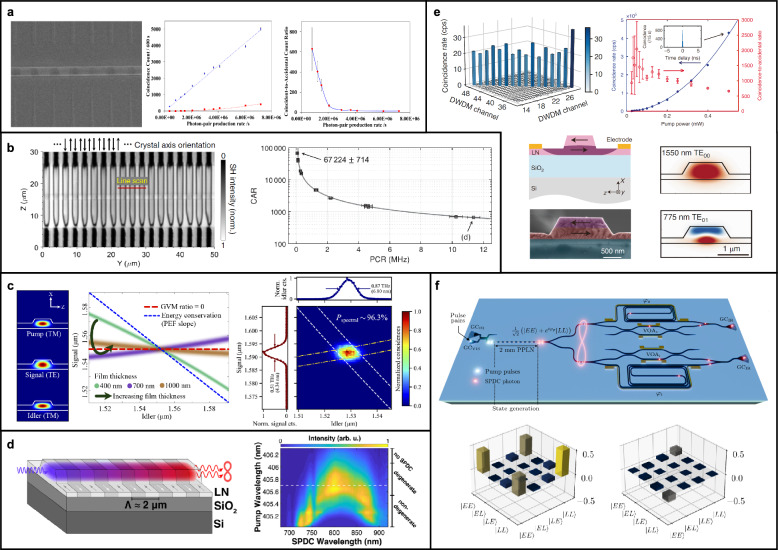
Entangled photon pair generation. **a** Photon-pair generation in an X-cut PPLN waveguide, demonstrating a high CAR. Reproduced with permission [134]. Copyright 2019, Optica Publishing Group. **b** Enhanced photon-pair generation performance achieved in a 5 mm X-cut PPLN waveguide, exhibiting ultrahigh CAR. Reproduced with permission [135]. Copyright 2020, APS. **c** Spectrally separable photon-pair generation in an optimized X-cut TFLN waveguide under type-II phase-matching conditions. Reproduced with permission [137]. Copyright 2022, Optica Publishing Group. **d** Photon-pair generation in the NIR spectral region for quantum applications in silicon-compatible wavelength ranges. Reproduced with permission [49]. Copyright 2024, Optica Publishing Group. **e** Photon-pair generation using innovative layer-poled waveguide geometry on TFLN, achieving high-quality energy-time entangled photon-pairs. Reproduced with permission [50]. Copyright 2024, Springer Nature. **f** Generation of time-bin entangled photon pairs with high fidelity and substantial on-chip brightness. Reproduced with permission [142]. Copyright 2024, Springer Nature

Exploiting the inherent nonlinearities of TFLN, which can occur even at the single-photon level, photon-pair generation was demonstrated in an X-cut TFLN with a CAR of $\sim$ 631 at a photon-pair production rate of $0.8\times 10^6/\hbox {s}$ in 2019 (Fig. 4a) [134]. In this work, a broadband difference frequency generation spanning 4.3 THz was also observed, enabling the generation of correlated photon pairs across multiple frequency channels via SPDC. In the same year, a CAR of 6900 in a 300 $\mu m$ long X-cut TFLN waveguide was reported, which demonstrated a broad signal-idler pair generation bandwidth of 120 nm with a SHG conversion efficiency close to $\sim 2,000\%\hbox {W}^{-1}\hbox {cm}^{-2}$. [139]

The following year, a CAR of 67,224 at a photon-pair coincidence rate of 76 kHz was achieved in a 5 mm X-cut TFLN device (Fig. 4b) [135]. This study also highlighted a significant improvement in the product of CAR and photon-pair coincidence rate, with a CAR of 668 observed at an increased photon-pair coincidence rate of 11.4 MHz. Notably, a heralded second order autocorrelation, $g_H^{(2)}$, of 0.022 was achieved, indicating high purity of generated photon-pair. Furthermore, this research demonstrated excellent two-photon interference visibility exceeding 99%, which is a clear evidence of energy-time entanglement. In the same year, in a Z-cut TFLN with a resonator configuration, photon-pair generation rates ranging from 8.5 MHz to 36.3 MHz were achieved at low pump power of 3.4 - 13.4 $\mu \textrm{W}$, resulting in CAR values exceeding 100, with some reaching $1.4\times 10^4$ [140]. With a low $g_H^{(2)}$ ranging from 0.008 to 0.097 with $\mu \textrm{W}$ pump power, this impressive performance underscore the potential for high-efficiency and high-purity photon-pair generation at low power levels. This remarkable performance was enabled by a triply resonant microring resonator, where all interacting waves were confined to low-loss fundamental modes with near-perfect spatial overlap, and nonlinear interactions occurred via the largest tensor component of LN’s second-order susceptibility.

In 2021, photon-pair generation with an extraordinary 100 THz (1.2 $\mu$m–2 $\mu$m) bandwidth was achieved through dispersion engineering, setting a new record [136]. This achievement was accompanied by an efficiency of 13 GHz/mW and outstanding noise performance, with a CAR exceeding $10^5$. Furthermore, the study confirms strong energy-time entanglement, with over 98% visibility in two-photon interference. In the same year, X-cut straight TFLN device achieved an ultrahigh generation rate of up to $2.79\times 10^{11}$ Hz/mW, and demonstrated eight-channel wavelength multiplexing with a spectral brightness of $\sim$ $10^9$ Hz/mW/nm [141]. This result was made possible by engineering a small group velocity dispersion (GVD) within the type-0 QPM bandwidth of 160 nm, which supports efficient phase matching over a broad spectrum and enables simultaneous generation of photon pairs across eight distinct wavelength channels. Two-photon interference is observed across all channels, with visibilities exceeding 97%, indicating high-quality energy-time entanglement.

While earlier studies primarily focused on achieving bright photon-pair generation with high CAR, scaling to multiple SPDC sources for quantum interference experiments requires high-purity photon states [2]. High purity ensures strong indistinguishability between photons from independent sources, which is essential for preserving entanglement and achieving high-visibility interferences [2, 135]. In this context, precise dispersion engineering becomes crucial, as it enables spectral separability and minimizes frequency correlations within the generated photon pairs, thereby supporting the scalability of quantum photonic architectures.

In 2022, optimizing the X-cut TFLN waveguide geometry to better control dispersion characteristics enabled the spectrally separable photon-pair generation in an X-cut device with type-II phase matching conditions (Fig. 4c) [137]. Spectral separability up to second order in phase mismatch can be achieved by engineering the GVM such that the pump group velocity lies between those of the signal and idler, or matches one of them. While this condition ensures separability in the joint spectral intensity (JSI), residual spectral phase correlations in the joint spectral amplitude (JSA) can be removed using standard pulse shaping techniques, provided that appropriate group-delay dispersion is applied to the pump before or within the waveguide. In the study, minimizing the correlation in the joint spectral intensity, unheralded second-order correlation function, $g^{(2)}$, measurements yielded a value of 1.86 for the signal photons, suggesting an estimated heralded-state purity of $\sim$ 86%.

Beyond photon-pair generation in the telecom band, recent studies have expanded into other wavelength regimes. In 2024, photon-pair generation at NIR wavelength was demonstrated (Fig. 4d), achieving a photon pair generation efficiency of 230 GHz/mW, a brightness of 1.6 GHz/mW/nm, and an impressive two-photon interference visibility of $\sim$ 100% [49]. This study marked the first successful energy-time entangled photon-pair generation experiments in the NIR wavelength range, opening up significant application potential in fields such as NIR imaging and spectroscopy.

Alongside correlated and entangled photon-pair generation in periodically poled TFLN waveguides, other innovative configurations utilizing MPM, layered geometries, and inverse design techniques on TFLN waveguides have been extensively explored [50, 61–63]. Notably, photon pairs generated in a layer-poled waveguide geometry demonstrated an impressive two-photon fringe visibility of 98%, indicating the generation of high-quality energy-time entangled photon-pair (Fig. 4e). These alternative approaches have proven to be highly effective in producing correlated and entangled photon pairs with superior brightness and efficiency, thereby expanding the versatility and potential of TFLN-based platforms.

While energy-time entanglement inherently arises from frequency correlations in SPDC, entanglement in other degrees of freedom, such as time-bin, polarization, or spatial modes, is equally important for scalable quantum information processing. This is because photons entangled only in frequency are generally less amenable to interference-based operations across different frequency modes. Time-bin entanglement, in particular, offers robustness against decoherence in fiber-based systems and compatibility with temporal multiplexing schemes.

In 2024, time-bin entangled photon pairs were generated using a TFLN device, achieving an on-chip brightness of 242 MHz/mW and a fidelity exceeding 90% (Fig. 4f) [142]. Although the lack of chromatic dispersion optimization led to some visibility degradation, quantum interference with a visibility of approximately 78% was still observed, demonstrating a violation of the Bell inequality. Leveraging the intrinsically large EO bandwidth of lithium niobate, this work further highlights TFLN’s strong potential for implementing high-speed, fiber-integrated time-bin quantum communication systems.

Looking ahead, further research into generating entanglement across multiple degrees of freedom, such as polarization-time-bin hybrid entanglement or spatial-polarization entanglement, will be essential for increasing the flexibility and dimensionality of integrated quantum systems. Recent progress in this direction includes the generation of NOON states, the realization of correlated and entangled photon pairs in TFLN-based metasurfaces, and the pursuit of polarization-entangled states [143–146], highlighting the dynamic and rapidly evolving nature of this field.

#### Squeezed state generation

Squeezed states were first theoretically introduced as a new class of nonclassical light in quantum optics, and their experimental realization followed through four-wave mixing in atomic vapors and optical parametric down-conversion [147–149]. These states reduce quantum uncertainty in one quadrature below the shot-noise limit at the expense of increased fluctuations in the conjugate quadrature. Comprehensive reviews provide detailed accounts of their physics and implementations [150, 151].

Squeezed light has since become central to quantum technologies. In the field of quantum metrology, squeezed light has emerged as a powerful tool for enhancing the sensitivity of measurements by surpassing the standard quantum limit. Recent advances demonstrate applications of squeezed states in areas ranging from photodetector calibration to mechanical vibration sensing on scattering surfaces, and on-chip generation of nonclassical light [152–156]. In quantum information science, it underpins continuous-variable (CV) protocols including quantum teleportation, quantum key distribution, and cluster-state quantum computing [157–160]. These advances highlight the importance of realizing compact, chip-scale squeezed-light sources. TFLN, with its strong $\chi ^{(2)}$ nonlinearity, low propagation loss, and compatibility with QPM and dispersion engineering, offers a promising route toward this goal.

In this context, when coherent light propagates through a nonlinear medium, photon–photon interactions redistribute noise between quadratures: one quadrature can be squeezed below the shot-noise limit at the expense of increased fluctuations in the orthogonal quadrature. This phenomenon has been widely explored in precision sensing and quantum information, and in CV processing where information can be encoded into quadrature amplitudes. The following section reviews progress in generating squeezed vacuum states within TFLN waveguides.

In 2018, optical components for generating (periodically poled TFLN), manipulating (reconfigurable TFLN beam splitter), and detecting (balanced homodyne detection setup) two separable vacuum squeezed states were successfully integrated into a single Z-cut lithium niobate platform for the first time, achieving a squeezing level of 1.38 dB [161]. This work represents a significant milestone in integrated quantum photonics, demonstrating an integrated nonlinear photonic platform that enables on-chip generation, tunable manipulation, and direct measurement of nonclassical optical states.

**Fig. 5:**
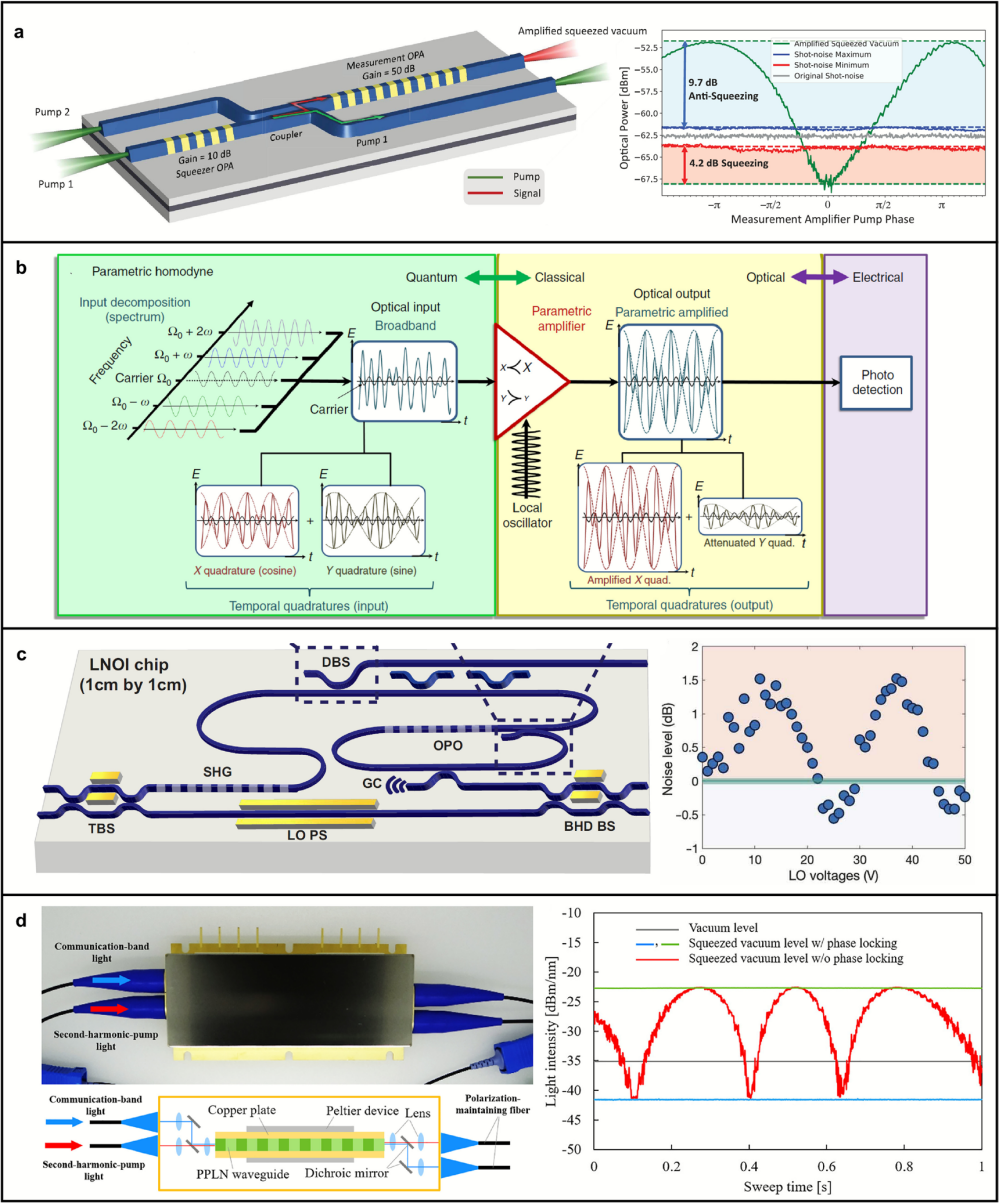
Squeeze state source generation. **a** Generation of vacuum squeezed states using two PPLN waveguide-based phase-sensitive OPAs. Reproduced with permission [164]. Copyright 2022, AAAS. **b** Schematic illustration of a parametric amplification-based all optical squeezing level measurement. Reproduced with permission [165]. Copyright 2018, Springer Nature. **c** CW single mode vacuum squeezed state generation incorporating BHD. Reproduced with permission [166]. Copyright 2024, AAAS. **d** Modularized TFLN waveguide for efficient vacuum squeezed state generation with high compatibility and usability by commercial fiber-coupled components. Reproduced with permission [169]. Copyright 2021, AIP Publishing

By 2022, a CW quadrature squeezing level of 0.56 dB was achieved in a Z-cut periodically poled TFLN nanophotonic device  [162]. The squeezer waveguide in this study exhibited a broad bandwidth of 7 THz, thanks to its single-pass configuration. In the same year, quadrature squeezing of 0.33 dB was realized in a similar setup with a picosecond pulsed pump [163]. The observed performance suggests on-chip squeezing levels as high as $-$1.7±0.4 dB. Importantly, this work demonstrates the potential of using short optical pulses at telecommunication wavelengths to define precise time-bin modes, which are highly advantageous for a wide range of CV quantum photonic applications. Notably, two consecutive X-cut periodically poled TFLN waveguide-based phase-sensitive OPAs were used to generate and measure a 4.9 dB vacuum squeezed state (Fig. 5a) [164]. Both OPA waveguides were pumped by a 75 fs few-cycle laser to generate the vacuum squeezed state and to scale the average photon number into the macroscopic regime, enabling all-optical measurement (Fig. 5b) [165]. The measured squeezing spectral bandwidth covered an extensive range of 25 THz.          

The following year, a CW single-mode vacuum with a 0.55 dB squeezing level was generated using an X-cut periodically poled TFLN-resonator-based parametric oscillator (Fig. 5c) [166]. In this study, the squeezer, periodically poled straight waveguide for generating SHG light, and tunable beam splitters for the balanced homodyne detection (BHD) setup were all integrated into the same TFLN device. In addition to periodically poled TFLN approaches, MPM-based vacuum squeezing with a level of 0.46 dB was achieved in 2024, demonstrating the potential for efficient squeezed state generation through a simple design and fabrication approach [167].

It is also noteworthy to mention that efficient vacuum squeezed state generation has been successfully achieved in a few micron-thick lithium niobate waveguides with geometry parameters approximately an order of magnitude larger than those in the TFLN platform. In such devices, 4.5 dB vacuum squeezing over a 2.5 Hz bandwidth, 6.3 dB vacuum squeezing over a 6 THz bandwidth, over-8 dB vacuum squeezing, and real-time 5 dB vacuum squeezing measurements over a 43 GHz bandwidth have been successfully demonstrated [168–171]. Furthermore, these devices have been modularized and packaged with commercial detectors and fibers, ensuring easy compatibility and usability (Fig. 5d) [168–171].

## Integrated photonic processors

Photonic quantum processor is the core of photon-based quantum information science, that can manipulate the quantum states of photons for computation and communication [172–175]. Constructing a photonic processor requires an array of optical components, including beam splitters and multiplexers for routing and distributing components, as well as modulators for optical phase control. Interferometric components, particularly Mach-Zehnder interferometers (MZIs) are fundamental in these processors, allowing coherent interference for programmable unitary transformation for quantum logic gates, state preparation, and quantum measurement [173, 176]. Fast modulation capability is crucial for photonic quantum processors, enabling high-speed dynamic control of quantum states, multiplexing, and feedforward operation [56, 177]. Furthermore, advanced components, such as polarization beam splitter and wavelength division multiplexers, enhance system flexibility by supporting polarization and spectral multiplexing, which are crucial for efficient multi-channel quantum systems.

Implementing the photonic quantum processor using free-space or fiber-based optics, such as programmable unitary transformations, presents significant practical challenges. In particular, each component, including beam splitter, phase shifters, and interferometric components, must be precisely aligned and stabilized for free-space optics, requiring a tight control over the path lengths and beam alignment. Even for the fiber-optical system, inherent sensitivity of the interferometers ultimately limit the stability of the fiber-system. This complexity grows rapidly with increasing numbers of modes, becoming unmanageable due to rapid growth in components and instability of the system [178].

Integrated photonics addresses these challenges by providing a robust platform where optical components can be patterned with high precision using standard nanofabrication, yielding reproducible, stable, and compact interferometric networks [178, 179]. Additionally, thanks to the dense integration of the optical components, implementation of large-scale interferometer become feasible [26, 173, 180]. Phase tuning within these integrated circuits can also be achieved through EO, thermo-optic, and acousto-optic modulations, just to name a few. As discussed in Background, TFLN is advantageous due to its strong Pockels effect, supporting the ultra-fast modulation exceeding over 10 GHz with low power consumption [45, 54, 55]. Although DC-drift of TFLN under DC bias presents stability challenges for EO modulation, feedback circuits may mitigate this problem [45, 54, 55]. Overall, the combination of speed, efficiency, and scalability makes TFLN highly attractive for ultra-fast photonic processing and quantum logic gate operations.

In this chapter, we present a comprehensive overview of the key photonic components required for scalable quantum information processing, with a particular focus on their implementation using TFLN platforms. We begin with programmable interferometric circuits, which form the backbone of linear quantum operations, and examine architectures that enable arbitrary unitary transformations. We then explore the design and performance of critical building blocks.

### Programmable interferometers for unitary transformation

Programmable interferometers are essential building blocks for implementing arbitrary unitary transformations in photonic integrated circuits. Large-scale quantum photonic systems depend on these transformations for state preparation, entanglement distribution and adaptive measurement. This section introduces the basic architectures and design principles of programmable interferometers. It also highlights the advantages of TFLN to realize such interferometers [173].

In a programmable unitary transformation, a *N*-mode input state $|\psi _\textrm{in}\rangle$ is transformed into an *N*-mode output state $|\psi _\textrm{out}\rangle$ through a unitary operation $\textbf{U}\left( \Phi ,\Theta \right)$, as expressed in Eq. 17:

17 $$\begin{aligned} |\psi _\textrm{out}\rangle = \textbf{U}\left( \Phi ,\Theta \right) |\psi _\textrm{in}\rangle \end{aligned}$$

where $\Phi$ and $\Theta$ represents sets of programmable parameters. On an integrated photonic chip, the transformation $\textbf{U}$ are typically realized through networks of programmable interferometers constructed from MZIs. The overall unitary transformation is achieved by cascading the transformation of individual MZIs, denoted as transformation $\textbf{T}$, as shown in Eq. 18:

18 $$\begin{aligned} \textbf{U}\left( \Phi ,\Theta \right) = \prod _{\left( m,n\right) \in S} \textbf{T}_{m,n}\left( \phi _{m,n},\theta _{m,n}\right) \end{aligned}$$

where *m* and *n* indicate the modes involved in interferences, while $\phi \in \Phi$ and $\theta \in \Theta$ are the phase shifts applied by two modulators. The sequence *S* defines the specific architecture of the MZI network.

**Fig. 6:**
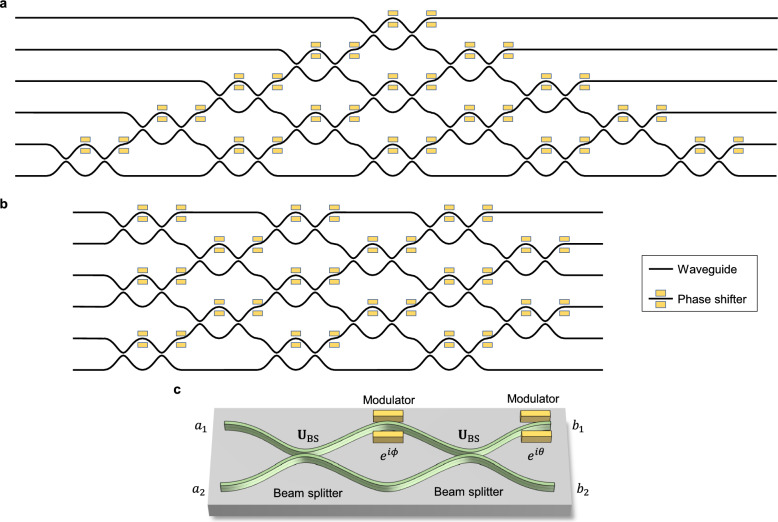
Architectures of programmable photonic interferometers. **a** Triangular (Reck) mesh design of a universal interferometer, implementing arbitrary unitary transformations using a sequence of tunable beam splitters and phase shifters arranged in a triangular layout [181]. **b** Rectangular (Clements) mesh design offering a more compact and symmetric structure while still enabling universal unitary transformations [182]. These structures are built on integrated photonic platforms using waveguides (black lines) and thermo-optic or EO phase shifters (yellow boxes). **c** Basic unit cell of the interferometers, comprising a tunable beam splitter and two phase modulators, realizing arbitrary 2 $\times$ 2 unitary transformations

Two representative architecture have been proposed to achieve arbitrary $N \times N$ unitary operations. In 1994, Reck et al. introduced a triangular mesh arrangements of MZIs, requiring of an optical depth of $2N-3$ for an $N \times N$ transformation, as depicted in Fig. 6a [181]. This configuration can lead to varying propagation lengths across different optical modes, making it sensitive to optical losses. Alternatively, in 2016, Clements et al. proposed a symmetric rectangular mesh configuration, reducing the optical depth of *N*, as displayed in Fig. 6b [182]. In this network, each optical mode interacts with its nearest neighbor early in the network. Thanks to the advantages of this configuration, such as the reduced optical depth, symmetric configuration, and high loss tolerance, it is commonly preferred choice for constructing arbitrary unitary transformation network.

Including the programmable unitary transform circuit, MZIs serve as the fundamental building blocks in various integrated photonic system that perform linear transformations. In integrated photonics, an MZI consists of two beam-splitting components connected by two optical waveguide arms, as displayed in Fig. 6c. A relative phase shift between these arms results in constructive and destructive interference at the output ports. By integrating phase-shifting components, such as thermo-optic or EO modulators, the interference condition can be dynamically tuned. The transformation provided by an MZI can be described as shown in Eq. 19:

19 $$\begin{aligned} \textbf{T}\left( \phi ,\theta \right) = ie^{i\frac{\phi }{2}}\begin{pmatrix} e^{i\theta } \sin {(\phi /2)} & e^{i\theta } \cos {(\phi /2)}\\ \cos {(\phi /2)} & -\sin {(\phi /2)} \end{pmatrix} \end{aligned}$$

where the $\phi$ and $\theta$ are the phase shift applied by the modulators, depicted in Fig. 6c.

As discussed before, entangled photon-pair generation based on nonlinear optical processes inherently exhibits probabilistic characteristics. This fundamental probabilistic nature poses challenges for constructing large-scale quantum photonic systems, where precise management of successful events becomes essential. In particular, interferometric-based unitary operations play a crucial role in scaling up quantum systems by enabling the generation of large-scale and multidimensional entanglement beyond weak nonlinearity of photons [180, 183]. An important example is fusion operations, which probabilistically combine smaller entangled photon states into larger cluster states, serving as critical resources for measurement-based quantum computing [184, 185]. Managing such probabilistic processes requires precise control over interferometers and unitary transformations to maximize the success of desired operations. Accordingly, adaptive circuits capable of dynamically reconfiguring in response to the success or failure of individual operations are critical for realizing scalable quantum photonic architectures [15, 177, 185, 186].

For example, refinery circuits have recently been introduced to overcome probabilistic nature of quantum photonic systems [15]. These refinery circuits implement adaptive spatial multiplexing using binary-tree arrangements of interferometers, to select the successful operations for further processing. This approach increases both success rates and enhances overall quality of the photonic quantum system, thereby improving the fidelity and throughput. Time multiplexing, utilizing fast modulation and delay lines, offers a promising avenue for implementing these adaptive circuits. By encoding information in time-bins and employing time-bin entanglement, it is possible to synchronize and combine photons from different time slots, effectively increasing the success probability of desired quantum operations. However, practical implementation of time multiplexing remains an area of research and has yet to be fully realized [58, 187].

While fundamental EO modulators and other basic components have been extensively demonstrated on TFLN due to their excellent EO properties and compact form factors, the large-scale integration of MZI arrays on TFLN platforms remains highly challenging, particularly in academic settings. Achieving low optical loss and uniform performance across many cascaded components is difficult even though several groups and foundries are actively working on it. Nevertheless, successful large-scale integration would greatly advance photonic quantum circuits, given the capability of TFLN for rapid modulation and its suitability for dense integration, which are crucial for realizing complex unitary transformations required in advanced quantum computing applications.

### Interferometric components on TFLN

Since the programmable interferometric components are important in the integrated photonics, various advances of the TFLN have been demonstrated. In the interferometric components, the beam splitting component is one of essential components, and the works on regarding that the fabrication tolerance, considering the scalable integrated photonics have been recently demonstrated. Also, the use of excellent EO modulation properties have made their advances, such as half wave voltage, modulation speed, and their applications.

#### Beam splitters

**Fig. 7:**
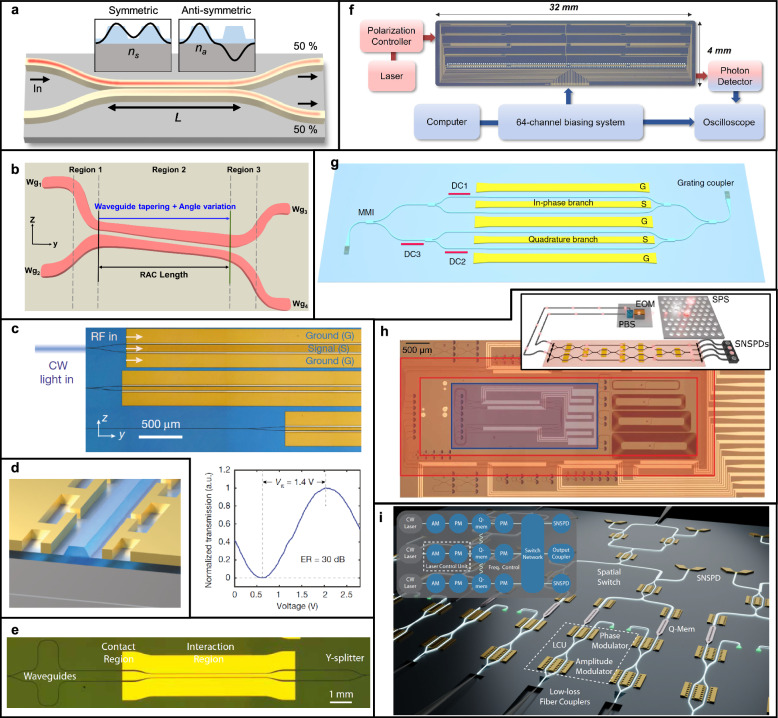
Research on fundamental building blocks for photonic and quantum information processing. **a** Schematic illustration of directional coupler, enabling power exchange by evanescent field coupling. **b** Rapid adiabatic coupler on Z-cut TFLN. Reproduced with permission [189]. Copyright 2025, Chinese Laser Press. **c** EO modulator demonstrating CMOS-compatible operation and ultra-broad bandwidth. Reproduced with permission [68]. Copyright 2018, Springer Nature. **d** EO modulator with segmented electrodes that reduce in microwave losses. Reproduced with permission [194]. Copyright 2021, Optica Publishing Group. **e** EO modulation spanning visible-to-NIR wavelengths, featuring sub-1 V$\cdot$cm voltage-length products. Reproduced with permission [197]. Copyright 2023, Springer Nature. **f** Integrated $4\times 4$ programmable photonic circuit using cascaded MZIs, offering enhanced scalability. Reproduced with permission [201]. Copyright 2023, APS. **g** Integrated coherent IQ modulators suitable for ultrafast coherent optical transmissions. Reproduced with permission [190]. Copyright 2020, Springer Nature. **h** EO modulation integrated with solid-state quantum dot emitters, enabling efficient photon routing and interference. Reproduced with permission [56]. Copyright 2023, AAAS. **i** NIR EO modulators developed for multiplexing quantum nodes. Reproduced with permission [57]. Copyright 2024, Springer Nature

Beam splitters are critical building blocks for interferometric circuits. A representative beam splitter is a directional coupler illustrated schematically in Fig. 7a. A directional coupler typically consists of two closely spaced parallel waveguides that exchange power through evanescent field coupling. The directional coupler has low insertion loss, but it is sensitive to fabrication tolerance. The length of directional coupler can be typically calculated as Eq. 20:

20 $$\begin{aligned} L = \frac{\lambda }{2(n_s-n_a)} \end{aligned}$$

where *L* is coupling length, $\lambda$ is operating wavelength, and $n_s$ and $n_a$ are effective mode indices of symmetric and anti-symmetric modes, respectively. The coupling strength and bandwidth can be determined and enables functionalities such as 50:50 power splitting.

Directional couplers on TFLN typically suffer from sensitivity to fabrication variations, causing performance deviations. For addressing this issue, Huang et al. demonstrated a directional coupler which operates with fabrication tolerant. Their design compensates for opposite changes in coupling induced by simultaneous width and gap fabrication errors, thus maintaining stable coupling performance despite fabrication variations exceeding ±100 nm [188]. Another recent work, demonstrated by Moon et al.(Fig. 7b), develops rapid adiabatic coupler through controlled tapering, curvature, and rotation to enable broadband low-loss, and balanced mode transition [189].

Complementing directional couplers, multimode interferometers (MMIs) represent alternative passive splitting components. MMIs exploit the Talbot effect within multimode waveguides to achieve compact and robust power splitting. Although typically associated with slightly higher insertion losses and back reflections compared to directional couplers, MMIs exhibit reasonable fabrication tolerance, and support multiple-output splitting configuration. Importantly, MMIs have been adopted in advanced TFLN photonic integrated circuits. For example, TFLN in-phase/quadrature modulators use 1$\times$2 MMI couplers as optical splitters and combiners for balanced operation [190]. Polarization-management circuits on TFLN have implemented Mach–Zehnder interferometers using 3-dB MMI couplers as the input and output beam splitters [191]. More recently, large-scale programmable microwave-photonic processors in TFLN incorporated MMIs as standard building blocks for multifunctional routing [192]. These demonstrations highlight that MMIs are not only feasible on TFLN but are already enabling diverse applications spanning modulators, polarization control, and reconfigurable processors.

#### Electro-optic modulation for tunable interferometers

EO modulation in TLFN emerged as a key enabling technology for advanced photonic processing and quantum information science due to its ultrahigh-speed modulation capabilities, low-voltage operation, and exceptional tunability. Significant progress in this field was first marked by Wang et al. in 2018, who demonstrated high-performance modulators (Fig. 7c) operating at CMOS-compatible half-wave voltages of 1.4 V [68]. In this work, microwave and optical fields are co-propagated using group-velocity matching with coplanar strip-line electrode, achieving ultra-low optical losses, and EO bandwidths of 210 GHz. In addition, Mercante et al. demonstrated high-speed modulators that push into the terahertz regime [193]. Their devices, featuring shallow rib waveguides and a precisely engineered index-matching strategy between the RF and optical modes, achieving 500 GHz bandwidth and half-wave voltage length product of 3.8 V$\cdot$cm. In 2021, Kharel et al. enhanced modulator performance by implementing micro-structured segmented electrodes (Fig. 7d), which reduced microwave losses and achieving minimal performance degradation even at frequencies surpassing 100 GHz [194].

The EO modulation capabilities on TFLN have also extended into the visible-to-NIR spectrum, which is the essential spectral range for quantum communications and computing. In 2019, Desiatov et al. introduced an ultra-low-loss integrated visible photonics platform with EO modulators operating with larger than 10 GHz bandwidth at visible wavelengths [195]. In 2023, Li et al. demonstrated MZI EO modulators for NIR and telecom band, which require low voltage-length product of 0.78 V$\cdot$cm and 1.29 V$\cdot$cm, respectively [196]. Renaud et al. expanded the application range of TFLN modulators into the visible-to-NIR spectrum. This work achieved a sub-1 V$\cdot$cm voltage-length product and low optical losses (Fig. 7e) [197]. Xue et al. demonstrated modulators with remarkable voltage-length products as low as 0.17 V$\cdot$cm at blue wavelengths, achieving unprecedented modulation efficiency across the visible spectrum with more than 20 GHz operation bandwidth [198].

Hybrid material platform and scalability remain critical for integrated photonics, enabling complex quantum processors through mature silicon-based photonic platforms [199–201]. Recent works addressed these issues by establishing wafer-scale hybrid TFLN modulators and multi-port programmable photonic circuits. In this direction, He et al. demonstrated hybrid silicon and lithium niobate MZI modulators in 2019, achieving low voltage-length products of 2.2 V$\cdot$cm, operating at an EO bandwidth of at least 70 GHz and supporting modulation rates up to 112 Gb/s [199]. In 2023, Ruan et al. successfully demonstrated a silicon nitride and lithium niobate heterogeneous integration platform, achieving high-performance EO modulators with half-wave voltages of 4.3 V, modulation bandwidths of 37 GHz [202]. Additionally, Zheng et al. successfully demonstrated $4 \times 4$ integrated programmable circuits using cascaded MZIs (Fig. 7f), achieving exceptionally low power consumption, high bandwidth, and ultra-fast switching times [201].

The broad range of applications enabled by TFLN-based EO modulation enhances both optical and quantum information processing. For example, Xu et al. demonstrated integrated in-phase/quadrature (IQ) modulators (Fig. 7g) that combine low drive voltage and ultra-broad EO bandwidth for achieving coherent optical transmission at data rate up to 320 Gb/s [190]. Hu et al. engineered on-chip EO frequency shifters and frequency domain beam splitters using coupled ring resonators [203]. This enable frequency conversion up to 28 GHz with conversion efficiency near 90 % and tunable splitting ratio. Sund et al. demonstrated a quantum processor that leverages EO capabilities to interface with solid-state quantum emitters (Fig. 7h) [56]. This device supports rapid on-chip photon routing and interference with visibilities over 90 %, addressing challenges in scalable photonic quantum computing. Most recently, in 2024, Assumpcao et al. realized a TFLN-based NIR platform specifically tailored for multiplexing of quantum nodes (Fig. 7i) [57]. This work utilizes EO modulators exceeding 50 GHz bandwidth with efficient frequency shifting over 50 % at 15 GHz for boosting entanglement distribution rates for quantum networks.

### Essential components for diverse quantum dimensions

Photons inherently possess multiple degrees of freedom, including wavelength, polarization, and spatial modes, each of which can be harnessed to encode, manipulate, and process quantum information. Exploiting these diverse degrees of freedom enables high-dimensional quantum information processing and enhances the scalability of quantum photonic systems. To fully leverage these capabilities, several essential components beyond interferometric elements are required. First, wavelength-division multiplexer (WDM) enable the encoding and routing of qubits across the multiple wavelength channels, allowing for dense integration and parallel processing. This is particularly advantageous that leverage frequency-encoded qubits. Polarization control components, particularly the polarization splitter rotator (PSR), are important for controlling the polarization-encoded qubits, which can be utilized for the arbitrary polarization states. Another approach is multimode manipulation, which utilizes spatial modes as an additional degree of freedom for encoding and processing quantum information. These components can expand the degree of freedom of the controlling qubit state, making the versatile qubit processors.

#### Wavelength division multiplexers

Exploiting the wavelength degree of freedom is fundamental for scaling the capacity of optical communication systems and for enabling high-dimensional quantum information processing. Managing multiple wavelength channels on a chip allows for parallelization of information streams and the generation of entangled states across different spectral modes. WDMs separate or combine multiple wavelength channels within communication systems, dramatically increasing data capacities. The common implementations of WDMs are arrayed waveguide gratings (AWGs) [55, 204–206] and multimode waveguide gratings (MWGs) [207, 208]. AWGs rely on a phased array of waveguides with varying lengths to create wavelength-dependent interference for wavelength separation. In contrast, MWGs utilize Fabry-Perot interferences using gratings in multimode waveguides to achieve compact spectral filtering. Recent advances in TFLN-based WDM devices have enabled enhanced performances including insertion losses and inter-channel crosstalk, making them increasingly relevant not only for classical photonic systems but also for emerging quantum applications.

**Fig. 8:**
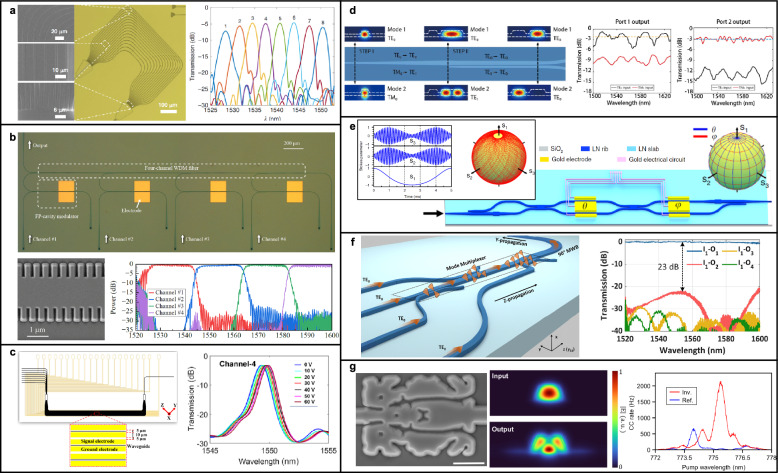
Essential building blocks for utilization of diverse quantum dimensions. **a** WDM using anisotropy free AWGs on X-cut TFLN. Reproduced with permission [205]. Copyright 2024, Springer Nature. **b** Ultra compact multimode waveguide grating-based WDM demonstrating efficient wavelength separation for dense spectral multiplexing. Reproduced with permission [207]. Copyright 2023, Light Publishing Group. **c** Electro-optically tunable AWG capable of dynamic wavelength adjustment, achieving tuning efficiency of 10 pm/V. Reproduced with permission [55]. Copyright 2025, AIP Publishing. **d** Broadband adiabatic PSR on TFLN, providing a broad operation bandwidth with minimal polarization crosstalk. Reproduced with permission [211]. Copyright 2021, Chinese Laser Press. **e** Active polarization management device integrating PSRs and EO modulation. Reproduced with permission [191]. Copyright 2022, Springer Nature. **f** Multimode multiplexing device on TFLN, employing anisotropic waveguide structures and adiabatic directional couplers. Reproduced with permission [219]. Copyright 2023, Wiley-VCH. **g** Inverse-designed compact TFLN mode converter for efficient photon-pair generation. Reproduced with permission [63]. Copyright 2025, De Gruyter

Yu et al. demonstrated an etch-less AWGs, enabling 16-channel division with low insertion loss and crosstalk for telecom and near visible wavelengths [204]. Addressing anisotropic challenges of X-cut TFLN, Yi et al. developed anisotropy-free AWGs on X-cut TFLN, achieving improved insertion loss of 2.4 dB and crosstalk of $-$24.1 dB (Fig. 8a) [205]. Otherwise, Liu et al. reported an WDM chip based on ultra-compact MWG with 20 nm wavelength spacing and low inter-channel crosstalk (Fig. 8b) [207]. Extending MWG-based approaches in 2024, He et al, realized a 12-channel LWDM device with remarkably low crosstalk (<−40 dB) suitable for O-band communications [208]. Wang et al. demonstrated a low-loss 8-channel AWG fabricated by photolithography-assisted chemo-mechanical etching, improving significantly upon conventional etching methods with 25 dB loss, while enabling smoother waveguide edges and lower propagation loss [206]. Subsequently, the same group introduced EO tunability into their AWGs (Fig. 8c), achieving dynamic wavelength tuning with 10 pm/V [55]. Additionally, Han et al. integrated angled MMI on erbium-doped TFLN for low-loss (< 0.7 dB), broadband (> 20 nm) spectral multiplexing, suitable for integrated amplification [209].

For scalable quantum technologies, WDM components should meet stringent performance requirements. Insertion losses should ideally be maintained well below 1 dB per channel to preserve photon  [15], and inter-channel cross-talk should be suppressed beyond -30 dB to avoid spectral contamination and ensure channel distinguishability. While recent TFLN-based WDM devices have demonstrated performance approaching or even exceeding these metrics, maintaining such low-loss and low crosstalk operation consistently across many channels remains an open challenge for large-scale quantum integration.

#### Polarization control

On-chip polarization management is essential for integrated photonic systems, playing a critical role in both optical communications and quantum technologies. PSRs are key devices that enable polarization state management by manipulating polarization-encoded quantum states as well as classical optical signals. Typically, in a PSR, TE-polarized incident light maintains its polarization state and transmits through the original waveguide path, whereas TM-polarized incident light rotates its polarization state and then transmits to the waveguide of another port, thereby splitting the path. Recent improvements in PSRs on TFLN platforms have demonstrated advances in adiabatic operation, polarization extinction ratio (PER), and operational bandwidth.

In 2021, Wang et al. reported a PSR using mode evolution that combined an adiabatic taper with asymmetric Y-junction [210]. This device achieved a PER larger than 26.6 dB and 19.6 dB for TE and TM polarization, respectively, in the 1520 - 1580 nm range. In the same year, Chen et al. demonstrated a broadband adiabatic PSR with measured bandwidth of 130 nm and polarization crosstalk of −10 dB (Fig. 8d) [211]. In 2022, Lin et al. advanced the polarization management with EO modulation (Fig. 8e), including PSRs with low losses and PER larger than 40 dB [191]. This device enables ultrafast and polarization control beneficial for quantum applications. In 2023, Shen et al. presented a broadband PSR design, employing conversion-enhanced multi-stage adiabatic tapers and directional couplers, enhancing the bandwidth of 160 nm and shorten the length [212]. In 2024, Zhang et al. enhance the compactness of PSR with a length of 61 $\mu$m using asymmetric directional coupler and taper [213]. Wang et al. introduced MMI-based PSR, which eliminated the stringent sub-micro gap requirements in directional coupler-based PSRs [214], with PER > 16 dB. In 2025, Li et al. demonstrated a broadband and fabrication-tolerant PSR that utilize stimulated Raman adiabatic passage, with PER exceeding 20 dB and operation bandwidth over a 130-nm band [215].

#### Multimode manipulation

Multimode manipulation also plays a critical role in both classical photonic signal processing and quantum information science. In classical system, it enables higher data throughput, spatial-division multiplexing, compact, and multifunctional photonic circuits [216]. In quantum domain, control over multiple mode unlocks high-dimensional encoding of photonic qubit, spatial-mode entanglement, and complex on-chip quantum interference, which are essential for future quantum networks and processors [217]. Recent TFLN-based devices for multimode manipulation mainly focused on the multiplexing and generation of high-order modes within the compact devices.

Yu et al. proposed multimode (de)multiplexers employing bound states in the continuum (BIC) on an etchless TFLN platform, effectively simplifying fabrication while maintaining minimal mode crosstalk (<$-$9.5 dB) over a broad bandwidth (70 nm) [218]. In 2023, Zhao et al. demonstrated anisotropic waveguide-based mode multiplexers that consist of adiabatic directional couplers with Z-propagation waveguides for avoiding mode hybridization and sharp multi-mode waveguide bend, enabling high-efficiency multimode waveguide integration (Fig. 8f) [219]. This device achieves low minimal loss (<0.2 dB) and low crosstalk (<−19 dB) over telecom band. By early 2024, Zhang et al. achieve compact TE-mode converters ($\sim$ 10 $\mu$m length) with low insertion losses (0.4$-$0.6 dB) and broad bandwidths (>100 nm) using shallowly etched waveguides and assisted-slot structures  [220]. Kwon et al. introduced inverse-designed TFLN mode converters optimized for quantum photonics applications, significantly reducing footprints ($3\times 15\,\mu \hbox {m}^2$) and achieving improved modal phase-matching and photon-pair generation efficiencies [62]. Extending this inverse-design methodology, Kim et al. further shrank device size ($5.6\times 7.6\,\mu \hbox {m}^2$) and enhanced mode purity (84.58%) and nonlinear efficiency, demonstrating substantial improvements in second harmonic generation and photon-pair generation (Fig. 8g) [63].

## Interface and detectors

In this chapter, we present a focused review of quantum interfaces and on-chip quantum detector integration strategies within TFLN platforms. In quantum photonic systems, one of the most critical challenges is optical loss. Due to the inherently weak nonlinearity of photons, most photonic quantum information processing schemes rely on measurement-based architectures rather than deterministic gate-based approaches. In such systems, single-photon detection plays a central role—not only in implementing quantum gates but also in verifying entangled states and enabling feedforward operations. As a result, loss at the interface and detection stages is particularly detrimental, directly degrading fidelity and increasing error rates. Every undetected photon represents lost quantum information, making high-efficiency coupling and detection essential requirements for building advanced quantum photonic systems.

We first examine key approaches for realizing low-loss fiber-to-chip optical coupling, which supports chip-to-chip and chip-to-detector connectivity and enables hybrid quantum architectures composed of high-performance fiber components and integrated photonic chips. We then explore the integration of quantum-compatible photodetectors, focusing on device architectures, materials, and fabrication techniques that enable highly efficient on-chip detection of quantum states. Particular attention is given to superconducting nanowire single-photon detectors (SNSPDs) and avalanche photodiodes, which serve as the workhorses for detecting CV and discrete-variable (DV) quantum states, respectively.

### Coupling strategies for low-loss TFLN platform

In integrated photonic systems, the interface between the optical fiber and the photonic chip is one of the most significant sources of optical loss [221]. Efficient fiber-to-chip coupling is essential for both classical and quantum applications, as it directly affects overall system performance. This issue is particularly critical in high-index-contrast platforms such as TFLN, where optical modes are tightly confined in submicron waveguides. The mode size mismatch between standard single-mode fibers (mode field diameters typically $\sim$ 10 $\mu$m) and the waveguides ($\sim$0.5–1 $\mu$m) results in poor spatial overlap and high insertion loss, often exceeding 5 dB per facet without optimization [222].

While minimizing insertion loss is one of the most critical factors, efficient fiber coupling indeed plays a pivotal role in addressing several fundamental challenges in scalable quantum photonic architectures. First, due to the finite size limitations of individual photonic chips, large-scale systems inevitably require interconnecting multiple chips, where low-loss fiber links become critical. Second, in DV quantum systems, SNSPDs necessitate cryogenic operation. Achieving highly efficient fiber coupling allows room-temperature photonic chips to interface seamlessly with cryogenic detectors, enabling hybrid systems without requiring the entire chip to operate at low temperatures. Third, optical fibers naturally serve as excellent delay lines, which is essential for encoding quantum information in the time-bin degree of freedom and for implementing temporal multiplexing schemes that enhance photon generation rates. Consequently, fiber-to-chip coupling is not merely a classical optical issue but a foundational requirement for scalable and versatile quantum photonic processors. Therefore, carefully engineered low-loss coupling interfaces in the TFLN platform are required for quantum information science.

#### Fiber-chip edge coupler

Fiber-chip edge couplers serve as one of the most effective methods, enabling direct horizontal coupling of light from the cleaved end of a fiber into the chip’s waveguide. As TFLN PICs continue to evolve for applications in communications, sensing, and quantum computing, edge couplers remain an essential component for high-efficiency fiber-to-chip interfaces.

A key challenge lies in the mode size mismatch between the optical fiber and the sub-micron waveguides in TFLN platform. To bridge this gap, mode size converters or inverse tapers are often incorporated into the edge coupler design to expand the optical mode in the waveguide, allowing for more efficient mode-field overlap with the fiber.

**Fig. 9:**
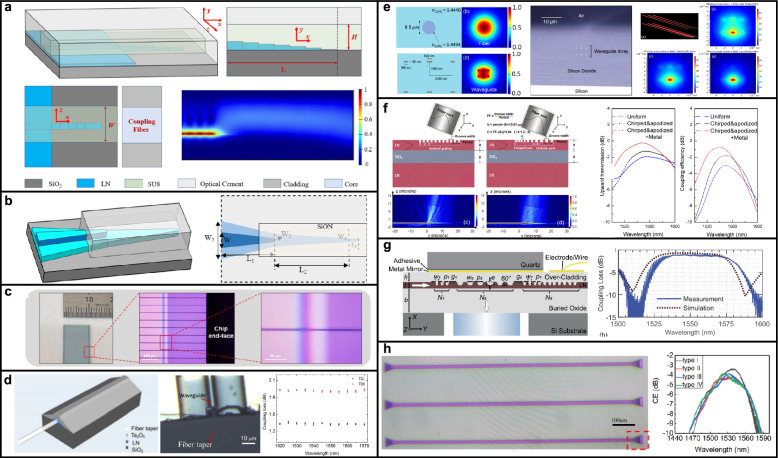
Coupling strategies for low-loss TFLN platform. **a** Edge coupler with SSC. Reproduced with permission [223]. Copyright 2025, MDPI. **b** Edge coupler with bilayer inverse tapered SSCs. Reproduced with permission [224]. Copyright 2023, SPIE. **c** Edge coupler with 3D mode size converters. Reproduced with permission [225]. Copyright 2025, Optica Publishing Group. **d** Ultra-low loss LN waveguide with an adiabatically tapered single mode optical fiber [227]. Copyright 2020, Optica Publishing Group. **e** Edge couplers utilizing multi-layer waveguide arrays with inverse-taper geometries [230]. Copyright 2021, Optica Publishing Group. **f** Chirped and apodized grating coupler with an optimized metal reflector. Reproduced with permission [242]. Copyright 2020, Optica Publishing Group. **g** Grating coupler with a cavity-assisted grating structure and a top metal mirror [245]. Copyright 2022, AIP Publishing. **h** Fabricated grating coupler with inverse design method. Reproduced with permission [247]. Copyright 2024, IEEE

Bao et al. demonstrated a stepped spot size converter (SSC) with a specific outer envelope profile to gradually transition the mode field diameter. By optimizing the envelope profile with a cosine-shaped curve, coupling efficiency (CE) was significantly improved. The maximum mode CE reached up to 95.35% when coupled with a single-mode fiber with a mode field diameter of 9.8 $\mu$m (Fig. 9a) [223]. Liu et al. presented the bilayer inverse tapered SSC approach integrated with the TFLN modulator, achieving high CE. By carefully designing the taper dimensions and materials, mode-field matching between the waveguide and the optical fiber was enhanced, resulting in the improved coupling performance (Fig. 9b) [224].

Jang et al. demonstrated 3D mode size converters using three-dimensional structuring techniques, enabling efficient edge coupling by tuning a mode size on the TFLN platform. An etching process employing a silicon external mask allowed for precise control over both height and width variations, facilitating effective mode transformation. Experimental results have shown edge CEs of approximately 1.16 dB/facet for the TE mode and 0.71 dB/facet for the TM mode at a wavelength of 1550 nm (Fig. 9c) [225].

Mode-size converters and inverse tapers in the aforementioned approaches typically require highly sophisticated fabrication processes. A comparatively simple and direct method to improve CE is to employ the single-mode fiber (SMF) itself as a spot-size reducer. The mode field diameter (MFD) of an SMF can be reduced by techniques such as fiber pulling, melting, or chemical etching  [226, 227]. Building on the on-chip mode expansion provided by inverse tapering, CE can be further enhanced through the use of etched fibers, in which the fiber cladding is locally thinned to decrease the MFD and achieve improved overlap with the expanded on-chip mode [228, 229]. The combination of inverse tapering and etched-fiber coupling maximizes the mode-overlap integral, relaxes the requirement for extremely narrow taper tips ($<100$ nm), and increases alignment tolerance, thereby supporting scalable and robust packaging of quantum and nonlinear photonic devices. Yao et al. demonstrated a highly efficient fiber-to-chip coupling technique by adiabatically tapering a standard single-mode fiber to match the mode field of a low-loss TFLN ridge waveguide, achieving simulated coupling losses as low as 0.32 dB (TE) and 0.86 dB (TM). Experimentally, they report 1.32 dB (TE) and 1.88 dB (TM) per-facet losses over a 50 nm bandwidth without AR coatings, highlighting a scalable and cost-effective approach for TFLN-based PIC applications (Fig. 9d) [227].

More recently, edge couplers utilizing multi-layer waveguide arrays with inverse-taper geometries have attracted increasing attention as highly promising candidates for efficient, fiber-array-compatible coupling interfaces [230]. By vertically stacking and laterally arranging multiple tapered waveguides, these couplers can provide enhanced mode matching and tolerance to alignment errors while supporting multi-channel operations (Fig. 9e). Such architectures are particularly appealing for large-scale quantum and classical photonic integration. However, their realization on lithium niobate platforms remains largely unexplored, representing an important direction for future development.

#### Grating coupler

Grating couplers have become indispensable in modern photonics by efficiently interfacing optical fibers with photonic integrated circuits [231–233]. Compared to an edge coupling that requires post-fabrication processes such as high-quality facet polishing and high-resolution optical alignment, a grating coupler that can be placed anywhere on a chip eliminates the need for the stringent post-fabrication processes and facilitates wafer-scale testing of photonic integrated circuits by enabling light coupling at any location on the chip. However, they typically exhibit higher insertion loss of 3–6 dB in the standard designs [234], narrow optical bandwidths, and polarization sensitivity, which can degrade performance in broadband or polarization-diverse systems. In fact, grating couplers on TFLN platform require precise control of etch depth, duty cycle, and sidewall angle to achieve optimal diffraction efficiency, and small deviations significantly degrade coupling performance. The RIE of lithium niobate is notoriously challenging, often leading to rougher sidewalls and reduced reproducibility compared to CMOS-compatible silicon platforms [235–237]. Moreover, achieving wafer-scale uniformity and apodized or chirped grating patterns is more difficult in TFLN due to its limited process maturity [238]. Current state-of-the-art TFLN grating couplers typically report higher losses per facet than those achieved in silicon or silicon nitride with optimized apodization and back-reflector designs [239, 240]. Therefore, advanced grating coupler technology has been devoted to enhancing the device performance [234, 239, 241].

A grating coupler specifically designed for the TFLN platform is an essential optical interface that efficiently couples light from optical fibers into TFLN waveguides [242–245]. Significant efforts have been dedicated to chirped and apodized structures [242, 246], inverse design [244, 247], and bottom reflector layer [243, 248], enabling high-efficiency grating coupler with enhanced CE, broad-bandwidth and polarization-insensitive operation. In 2015, a grating coupler on the TFLN platform was firstly reported [249]. In the early stages of research, the CE of grating couplers was not that high, making them not suitable for quantum integrated circuits [249, 250].

Recent advancements have focused on optimizing grating structures, refining etching methods, and developing innovative apodization techniques to improve CE and bandwidth. The chirped and apodized grating couplers achieved CE of $-$6.9 dB. The CE was further improved up to $-$5.5 dB via a metal reflector inserted between the oxide layer and lithium niobate substrate (Fig. 9f) [242]. Chen et al. also reported an impressive coupling loss as low as $-$0.89 dB at telecom wavelengths using cavity-assisted grating structures with reflective metal layers(Fig. 9g) [245]. This advancement represents one of the highest efficiencies achieved in the TFLN platform to date. He et al. presented the inverse designed grating coupler with CE of $-$3.9 dB, enabling balanced CE and the broadband 3-dB bandwidth of 90 nm (Fig. 9h) [247].

### Integrated on-chip detectors

Integrated on-chip detectors are essential components in the development of scalable and efficient quantum photonic technologies, playing a pivotal role in the measurement and analysis of quantum states. In quantum photonics, the measurement process is not only a means of information extraction but also an active part of quantum state manipulation and verification.

The integration of single-photon detectors (SPDs) onto the TFLN platform is of paramount importance in advancing quantum technologies, leading to enhanced performance and scalability in quantum applications. The various technologies for single-photon detection have been eagerly investigated with SNSPD, single-photon avalanche diodes (SPAD), Balanced Photo-Detectors (BPD), photon-number-resolving detector (PNRD), photomultiplier tube (PMT), silicon photomultiplier (SiPM), and transition-edge sensor (TES) [251–257]. Particularly, integrated on-chip detectors such as SNSPDs and high-efficiency III-V based detectors are critical frontier in terms of detecting DV and CV quantum states in advancing quantum technologies. In general, both paradigms offer different trade-offs. DV systems excel in robustness against noise and are more straightforward for encoding qubits, but they face scalability challenges, especially in photon generation and detection. CV systems, on the other hand, benefit from deterministic state preparation and scalability due to the maturity of telecom-band photonics, but they often require ultra-low loss photonic systems and highly squeezed states  [258].

Detection schemes for DV and CV quantum systems differ fundamentally, reflecting the nature of the quantum information they encode. In DV systems, quantum states are typically detected using mainly SPDs such as SNSPDs and SPADs. These detectors provide click/no-click binary outcomes, making them suitable for qubit-level measurements like those needed in QKD and photon-counting-based quantum logic operations [259–261]. In contrast, CV systems rely on measuring continuous quadratures of the electromagnetic field, which is accomplished through homodyne detection using high-efficiency III-V based photodetectors. While homodyne detection is the primary method for measuring CV quantum states, photon-subtraction techniques, which are essential for generating non-Gaussian quantum states in CV systems, require single-photon detection capabilities. Such operations typically rely on SPDs like SNSPDs or PNRDs such as TESs, highlighting the complementary role of SPDs or PNRDs even within CV quantum protocols.

#### Waveguide-integrated SNSPDs

While efficient fiber-to-chip coupling enables the use of fiber-coupled SNSPDs as a golden standard for high-performance detection in conventional quantum photonic systems, waveguide-integrated SNSPDs provide a complementary approach that offers several unique advantages. Integrating SNSPDs directly onto the photonic chip enables high detection efficiencies, broadband operation, and more compact system architectures [262, 263]. Moreover, in specific quantum optical systems where fast communication between photonic circuits and electronic control is critical, waveguide-integrated SNSPDs can offer significant benefits by minimizing optical coupling interfaces and reducing latency [263]. In this section, we review recent developments in waveguide-integrated SNSPDs on the TFLN platform, highlighting key advances in materials, device performance, and integration strategies.

Integration SNSPDs directly onto photonic chips have emerged as a prominent technology for detecting DV quantum states in scalable and compact quantum photonic circuits, encoding qubits in well-defined degrees of freedom of single photons, such as polarization, time-bin, or spatial mode. Accurate readout of these quantum states requires detectors with high efficiency, low dark counts, and precise timing resolution-all of which are characteristics of SNSPDs [251, 264].

**Fig. 10:**
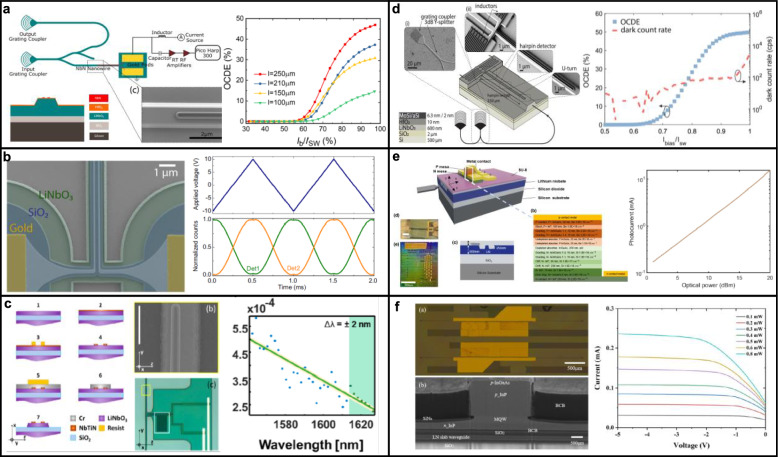
Integrated on-chip detectors. **a** Integration of niobium nitride (NbN)-based SNSPD on TFLN platform, achieving high-performance OCDE in accordance with the nanowire length. Reproduced with permission [81]. Copyright 2020, AIP Publishing. **b** Colored SEM image of waveguide-integrated SNSPD using NbTiN material, achieving high-performance EO modulator. Reproduced with permission [82] Copyright 2021, Springer Nature. **c** Integration of NbTiN-based SNSPDs onto 300 nm TFLN waveguides, showing the ability to extract the wavelength of detected photons. Reproduced with permission [265]. Copyright 2023, ACS Publications. **d** Heterogeneously integrated molybdenum silicide SNSPD onto TFLN waveguide and on-chip detection efficiency. Reproduced with permission [266]. Copyright 2024, ACS Publications. **e** MUTC photodiodes integrated on TFLN and their measured photocurrent results. Reproduced with permission [269]. Copyright 2022, Chinese Laser Press and Optica Publishing Group. **f** Heterogeneously integration III-V-on-TFLN photodetector with measured I-V curve. Reproduced with permission [270]. Copyright 2022, Optica Publishing Group

The recent research has demonstrated the successful monolithic integration of SNSPDs using NbN, NbTiN and MoSi materials onto TFLN platform. This enables low-loss, high-speed detection within a monolithic platform, reducing the complexity and inefficiencies of off-chip coupling. In 2020, integration of NbN-based SNSPD on TFLN platform was presented, showing an on-chip detection efficiency of 46% with high-performance of dark count rates and timing jitter at temperature of 1.7 K (Fig. 10a) [81]. Lomonte et al. demonstrated electrically tunable Mach-Zehnder interferometer and two waveguide-integrated SNSPDs at outputs. The main performance of integrated NbTiN-based SNSPDs showed on-chip detection efficiency of 24-27%, low dark count rates of $\sim$ 2 counts per second (cps), timing jitter of 17 ps, and dead time of $\sim$ 6 ns at a base temperature of $\sim$ 1.3 K (Fig. 10b) [82]. Prencipe et al. presented the integration of NbTiN-based SNSPDs onto 300 nm TFLN waveguides, exploiting the photon-energy-dependent responsivity of SNSPDs. A key feature is the ability to extract the wavelength of detected photons with an uncertainty as low as $\pm 2$ nm. This approach significantly reduced the device footprint ($\sim 300 \times 180 \mu \text {m}^{2}$), offering a scalable solution for quantum communication systems requiring dense wavelength multiplexing (Fig. 10c) [265]. In 2024, Colangelo et al. demonstrated heterogeneous integration of MoSi-based SNSPDs onto TFLN waveguides with a confomal hafnium oxide buffer layer. The MoSi-based SNSPD achieved on-chip detection efficiency of 50.2% and a timing jitter of 82 ps at a base temperature of 0.78 K, opening possibilities for monolithic quantum processors and cryogenic photonic-electronic hybrid systems (Fig. 10d) [266].

#### Integrated III-V semiconductor-based detectors

Integrated III–V semiconductor-based detectors can operate at significantly higher and more relaxed temperature conditions than SNSPDs, which require deep-cryogenic environments below $4\,\textrm{K}$ to achieve high detection efficiency and low dark counts. This relaxed requirement allows III–V detectors to function with simple thermoelectric or liquid-nitrogen cooling, offering practical advantages for scalable, room-temperature or moderately cooled quantum photonic systems. That is because operating TFLN devices at cryogenic temperatures introduces several difficulties, including temperature-induced index instability and thermal-expansion mismatch between various materials within packaged TFLN photonic chips. Such effects can degrade optical alignment as well as the efficiency of modulation and nonlinear interactions. [267, 268].

Heterogeneous integration of III–V-based photodetectors onto the TFLN platform represents a powerful approach to combine the unique advantages of both material systems for high-performance quantum photonic circuits. Extensive efforts have been devoted to integrating III–V photodetectors such as photodetectors, BPDs, and even SPADs onto TFLN platforms, leveraging their direct bandgaps and strong absorption at telecom wavelengths. Integration techniques including direct wafer bonding, adhesive bonding (e.g., BCB), flip-chip assembly, and transfer printing have been explored to achieve efficient evanescent coupling between the TFLN-guided mode and the III–V absorption region. A key benefit of this approach is that it allows co-integration of detection and modulation: TFLN-based phase shifters and interferometers can be combined with III–V detectors on the same chip, resulting in compact, low-loss, and multifunctional quantum photonic processors. The co-integration is particularly valuable in the telecom band (1.3–1.55 $\mu$m), where both TFLN and InGaAs-based detectors operate efficiently.

III–V semiconductor-based photodetectors are of particular importance in CV quantum photonic systems, since homodyne measurement is the core detection scheme in CV quantum information processing. It critically relies on high-speed and low-noise BPDs to measure quadratures of photons. Their high bandwidth, low dark current, and telecom compatibility directly determine the fidelity of on-chip quantum state reconstruction. In 2022, Guo et al. demonstrated the first heterogeneously integrated modified uni-traveling carrier (MUTC) photodiodes on the TFLN platform, achieving unprecedented performance in on-chip detection. The MUTC photodiodes with an InGaAs/InP epitaxial structure exhibit a record-high 3-dB bandwidth of 80 GHz, a low dark current of 3 nA, and an internal responsivity of 0.6 A/W at 1550 nm. The heterogeneous integration was realized using wafer-scale die bonding with SU-8 polymer and optimized evanescent coupling through a TFLN waveguide structure (Fig. 10e) [269]. Recently, Zhang et al. demonstrated the heterogeneous integration of III-V-based photodetectors on TFLN platform, presenting a responsivity of 0.38 A/W at 1540 nm and low dark current of 9 nA at $-$0.5 V (Fig. 10f) [270]. Recently, BPDs have also been demonstrated on the TFLN platform in recent work, demonstrating the feasibility of fully integrated homodyne detections [271].

III–V semiconductor-based detectors also hold strong potential for DV quantum photonic systems, where the development of high-performance SPADs is essential for realizing on-chip single-photon detection. While III–V SPADs offer the potential for high-speed and low-dark-count operation under relaxed cooling conditions [272], their heterogeneous integration onto the TFLN platform remains largely unexplored. Compared to SNSPDs, III–V SPADs generally exhibit lower detection efficiencies, higher dark count rates, and inferior timing resolution. Nevertheless, a key advantage of III–V SPADs lies in their relaxed cooling requirements. They can operate at or near room temperature without cryogenic systems, greatly simplifying system complexity and integration overhead. Continued advances in III–V SPAD design and heterogeneous bonding processes will be critical for achieving high-efficiency, low-noise, and moderately cooled operation, paving the way toward deployable DV quantum photonic systems based on TFLN platforms.

## Discussion and outlook

We have reviewed the current state-of-the-art in TFLN-based quantum photonic components, including quantum light sources, reconfigurable processors, and optical interfaces and on-chip detectors. Each section provided insight into the physical mechanisms, device architectures, and recent experimental advances that underpin TFLN’s potentials in integrated quantum photonics. While these developments have demonstrated impressive performance at the component level, most implementations remain focused on small-scale devices. To realize scalable and practical quantum photonic systems, it is crucial to transition from individual device demonstrations toward system-level integration and co-design.

In addition to sources, processors, and detectors, scalable quantum systems also require quantum memories to enable synchronization, buffering, and networked operation. Proof-of-principle demonstrations of quantum storage have been reported in rare-earth-doped lithium niobate waveguides fabricated by ion diffusion on bulk substrates, particularly in work by the Tittel group  [273]. However, comparable demonstrations in TFLN are still at a very early stage, with no large-scale or integrated implementations yet available. This underscores quantum memories as an essential but presently missing building block in TFLN-based integrated quantum photonic systems, and an important direction for future research.

Although we have structured our review around distinct building blocks-sources, processors, and interfaces/detectors-to reflect current status, practical quantum photonic circuits must ultimately combine these elements into fully functional systems. For example, the generation of complex entangled states required for fault-tolerant quantum computing [14, 185] will inevitably involve multiple synchronized single photon sources connected with actively reconfigurable photonic processors. To overcome the probabilistic nature of photonic quantum system, real-time feedforward operations must be enabled through integration of detectors with the processor circuitry [186, 187]. These examples underscore the need to move beyond component-level optimization and adopt system-level design strategies that address co-integration and overall scalability [15].

In such integrated systems, a single chip may need to incorporate dozens to hundreds of quantum light sources, modulators, and detectors, all of which should operate in a synchronized and phase-stable manner. These densely integrated photonic chips will also require connection to a large number of optical fibers, potentially reaching tens to hundreds of input/output channels per chip, to support multiplexed quantum information transfer and interfacing with other systems. Moreover, the quantum photonic circuitry must be tightly integrated with electronic control circuits to enable high-speed signal routing and real-time feedback and feedforward operations. This level of complexity highlights the importance of co-design strategies that consider photonic, electronic, and packaging aspects. Although the fabrication maturity of TFLN is not yet comparable to that of CMOS-based silicon photonics, rapid progress in LN processing, including high-quality etching, bonding, and periodic poling, together with ongoing efforts toward reliable wafer-scale manufacturing, is bringing the field ever closer to realizing fully integrated quantum photonic circuits with on-chip sources [69, 274].

Another essential ingredient for scaling up TFLN-based quantum systems is the development of advanced photonic interfaces and hybrid integration strategies. As quantum photonic circuits evolve toward more complex, fully integrated architectures, conventional coupling approaches such as fiber-edge or grating couplers may become limiting in terms of footprint, bandwidth, and loss. Especially, the monolithic TFLN faces serious challenges in scalability and yield. First, fabrication of high-quality etched features (gratings, waveguide sidewalls, inverse tapers) requires highly precise lithography, low sidewall roughness, uniform etch depths, and high aspect ratio control. Variations cause scattering loss and reduce coupling or modulation efficiency. Second, process compatibility (with foundry-level fabs) is less mature for TFLN than for silicon or silicon nitride, driving higher costs, more stringent thermal management, and lower throughput [275]. Advanced interface technologies enabled by hybrid or heterogeneous integration represent a significant leap forward in this regard. The hybrid or heterogeneous integration of TFLN onto established low-loss platforms like silicon or silicon nitride can leverage the complementary advantages of both approaches, combining the efficient EO behavior of LN with the low propagation losses, mature fabrication infrastructure, and dense routing capabilities of silicon-based platforms  [275, 276].

Although the integration of III–V based photodetectors on TFLN is still an emerging area, such heterogeneous integration approaches are expected to play a key role in enabling fully integrated, high-speed, and low-noise quantum photonic systems. Furthermore, in addition to integrating on-chip SPDs such as SNSPDs and III–V based materials capable of detecting CV quantum states, III–V based light emitters have recently been explored using a myriad of approaches including adhesive bonding, flip-chip bonding, wafer bonding, and transfer printing [270, 277–279]. These efforts pave the path toward fully integrated optical systems on the TFLN photonics platform.

Beyond these architectural considerations, a deeper understanding of the quantum functionalities enabled by TFLN is essential. In the remainder of this discussion part, we examine in detail the roles and implementation challenges of each key building block, including quantum state generation, entanglement schemes, multi-photon scalability, squeezed-light systems, and advanced interfacing technologies, from the perspective of realizing next-generation quantum photonic circuits based on TFLN.

First, we begin by discussing recent progress and open challenges in quantum light generation, with a focus on single-photon sources. For translating potential applications into practical real-world systems, substantial further research on quantum state generation is still necessary. In the context of single-photon generation through heralding of one photon in a photon pair, bright SPDC-based sources in TFLN waveguides have been actively investigated in the third chapter of quantum light sources. While these works have demonstrated high generation rates and excellent two-photon interference visibilities, experimental demonstrations of highly pure on-chip TFLN photon sources and on-chip multi-photon quantum light sources remain limited [46, 137]. Achieving such advanced sources requires overcoming several challenges. First, the design of optimized SPDC waveguides and precisely engineered poling structures is essential to suppress spectral correlations and enhance purity. This often necessitates long interaction lengths or carefully tailored pump pulse durations to achieve narrow joint spectral amplitudes. Additionally, generating near-identical heralded single-photon sources with minimal variation in brightness, spectrum, and temporal profile is critical for enabling high-visibility interference among multiple sources. Beyond the highly pure single-photon source development, integrated photonic circuits must include high-extinction filters-often require suppression levels exceeding $\sim$100 dB-to eliminate residual pump photons [14].

Two-photon entangled sources are foundational for quantum information science and the generation of entangled photon pairs via SPDC has long served as a practical and accessible approach for probing fundamental quantum phenomena [280–285]. While SPDC naturally generates frequency domain entangled signal and idler photons due to energy conservation, other degrees of freedom, such as polarization, time-bin, spatial mode, and orbital angular momentum, can also be harnessed for entanglement [286]. Among these, time-bin and polarization-entangled sources are in particular interest for quantum communication, quantum computing, and networking. At present, entanglement generation in TFLN waveguides has primarily focused on energy-time entangled states, leaving time-bin and polarization-entangled generation largely underexplored [142]. Given the widespread use of fiber-optic links in quantum information systems, time-bin entanglement may offer more stable and robust encoding for practical implementations. Furthermore, generating multiple time-bin entangled states across different time slots enables time-division or wavelength-division multiplexing, which can be harnessed to increase the capacity of quantum information and communication systems.

Beyond two-photon entangled sources, the generation of large-scale and multi-photon entangled states, such as cluster states, is a key requirement for fault-tolerant quantum computing based on DV encoding [185]. Cluster states serve as universal resource states for measurement-based quantum computation. However, their realization using photonic platforms is highly nontrivial, as it requires not only many entangled photons but also high fidelity and synchronization. In SPDC-based systems, which are inherently probabilistic, the success probability of generating *N*-photon states decreases exponentially with *N*. Moreover, higher order multi pair generation from a single source, particularly four photon and higher order contributions, becomes increasingly probable, introducing noise and limiting scalability.

To address this, multiple independent but indistinguishable heralded single photon sources must be operated simultaneously. Achieving this in a scalable way requires source multiplexing [187]. TFLN photonic platforms might be well-suited to implement such multiplexing schemes, thanks to their low-loss, high-speed EO modulation capabilities and high integration density. This platform supports the monolithic integration of high-purity SPDC sources, fast optical switches, delay lines, and active feed-forward control circuitry, which are all key components in multiplexed multi-photon generation. Another approach for scaling up cluster states involves entanglement fusion operations, where smaller entangled pairs are combined into larger states. These schemes are also inherently probabilistic and require additional ancilla photons and high-speed reconfigurable photonic circuitry to boost success probabilities [185, 287, 288]. Here again, TFLN offers key advantages enabling fast and low-power switching. These features enable the implementation of fusion gates with sufficient speed and phase stability for high-fidelity entanglement operations.

On the other hand, CV-based photonic quantum system often deploys the squeezed vacuum states as a fundamental resource for quantum information processing. Currently, squeezed light generation in TFLN platforms has primarily focused on single vacuum squeezer implementations [162, 164, 166, 168–170]. Despite their deterministic nature in generation process, squeezed states are highly sensitive to optical loss. Once the parametric gain is sufficiently high, the measurable squeezing level is ultimately limited by the total system loss. This makes loss minimization a critical factor in the design and integration of squeezed-light systems. In the case of TFLN, most of the low-loss demonstrations have been performed using unpoled waveguides [289]. However, periodic poling, which is essential for phase-matched nonlinear interaction, introduces domain inversions that, together with orientation-dependent etching chemistry, can increase propagation loss significantly. Although z-cut devices may exhibit slightly improved performance, the fabrication of low-loss PPLN waveguides remains an open and important challenge [290]. In addition, coupling loss at the fiber-chip or free-space-chip interface accounts for a large portion of the total system loss and must be addressed through improved packaging and interface engineering [14, 225, 245]. The same applies to detector integration, where high-efficiency and high-speed are necessary to maintain the fast characterization of the quantum states encoded in CV.

A key future direction lies in going beyond isolated squeezers to integrated circuits composed of multiple squeezing elements. While bulk-optic and micro-waveguide-based systems have already demonstrated record squeezing levels, they typically involve single-element configurations [152, 170]. In contrast, integrated arrays of coherently interfered squeezers on a single chip open a promising path toward scalable CV entangled states. This architecture could enable the generation of time-bin encoded cluster states for CV quantum computing, where low-loss delay lines, fast optical switches, and high-performance avalanche photodiodes are used together for large-scale quantum processing [291, 292]. Achieving such functionality on chip would mark a significant milestone for photonic quantum information science.

In parallel, the generation of non-Gaussian quantum states through photon subtraction from the squeezed vacuum states represents a critical frontier [293, 294]. While coherent states remain unchanged in their photon-number distribution when photons are subtracted, squeezed vacuum states undergo fundamental changes in their quantum statistics. This process is widely used for the generation of non-Gaussian state, and it is called photon-subtraction [295]. The photon-subtraction enables the creation of non-Gaussian states such as Schrödinger cat states. Furthermore, the cat states are considered as central ingredients for preparing Gottesman-Kitaev-Preskill (GKP) states, which are essential for fault-tolerant CV quantum computation [293]. TFLN offers strong advantages for realizing these goals when the photonic circuits are integrated with SNSPDs or PNRDs. Finally, circuits combining squeezers, interferometers, and detectors could support heralded or feed-forward controlled preparation of non-Gaussian states directly on chip, offering a path toward high-performance CV-based quantum photonic system.

Another important direction for future exploration is the generation of multimode squeezed states and entangled states in the frequency bin [286, 296–298]. Theoretical and simulation studies have shown that shaping the pump and engineering the domain structure in nonlinear media can enable highly structured entanglement in the frequency domain. Although such demonstrations have been explored mostly in bulk optics, their translation to integrated photonic platforms remains at an early stage [299, 300]. TFLN might be well positioned to enable compact, on-chip implementations of high-dimensional, frequency-multiplexed CV quantum systems. Realizing this capability would further expand the scope of what is possible in integrated quantum photonics.

In this review, we have highlighted recent advances in TFLN-based integrated quantum photonics, which include quantum light sources, reconfigurable processors, optical interfaces, and detectors. Although many of the demonstrations remain at the component level, the rapid pace of progress, both in device performance and integration capability, points to a promising future. With continued improvements in fabrication, loss reduction, and system-level integration, TFLN photonics are expected to serve as a scalable and versatile platform for next-generation quantum technologies. As the field moves forward, we anticipate that the unique strengths of TFLN will play a central role in bridging the gap between experimental quantum optics and practical, on-chip quantum information systems.

## Data Availability

Not applicable.

## Abbreviations

AWG: Arrayed waveguide grating
CAR: Coincidence-to-accidental ratio
CV: Continuous-variable
DFG: Difference-frequency generation
DV: Discrete-variable
EBL: Electron beam lithography
EME: Eigenmode expansion
EO: Electro-optic
FEM: Finite element method
FDTD: Finite-difference time-domain
FOM: Figure of merit
ICP-RIE: Inductively coupled plasma reactive ion etching
MMI: Multi-mode interference
MPM: Modal-phase matching
MWG: Multimode waveguide grating
MZI: Mach–Zehnder interferometer
NIR: Near-infrared
OPO: Optical parametric oscillator
PER: Polarization extinction ratio
PIC: Photonic integrated circuit
PMT: Photomultiplier tube
PNRD: Photon number resolving detector
PSR: Polarization splitter rotator
QKD: Quantum key distribution
QPM: Quasi-phase matching
SHG: Second harmonic generation
SFG: Sum-frequency generation
SiPM: Silicon photomultiplier
SNSPD: Superconducting nanowire single-photon detector
SPAD: Single photon avalanche diode
SPD: Single photon detector
SPDC: Spontaneous parametric down-conversion
TES: Transition edge sensor
TE: Transverse electric
TFLN: Thin film lithium niobate
THG: Third harmonic generation
TM: Transverse magnetic
WDM: Wavelength division multiplexing
